# Targeting Metabolic Pathways to Direct T-Cell Trafficking: Therapeutic Perspectives

**DOI:** 10.3390/biomedicines14092100

**Published:** 2026-09-17

**Authors:** Xiayinan Song, Shengxuan Zhao, Weiheng Zhang, Yan Wang, Fengjie Zheng

**Affiliations:** 1 School of Traditional Chinese Medicine, Beijing University of Chinese Medicine, Beijing 100105, China; 2 Second Affiliated Hospital, Heilongjiang University of Chinese Medicine, Harbin 150001, China

**Keywords:** T cell, trafficking, metabolic reprogramming, immunotherapy

## Abstract

Despite advances in immunomodulatory therapies, dysregulated T-cell trafficking persists as a pathological cornerstone in chronic inflammation, autoimmunity, and cancer. This spatially precise navigation—orchestrated by receptor–ligand cascades—ensures immune surveillance but drives disease when impaired. Targeting individual receptors faces limitations due to functional redundancy. Emerging research establishes metabolic reprogramming as a critical regulator of trafficking efficiency, where glucose, amino acid, lipid, and mitochondrial metabolism dynamically control all stages: from chemotaxis, selectin-mediated rolling, and integrin-dependent adhesion to transendothelial migration and interstitial migration. Critically, these pathways integrate energy supply, metabolite signaling, and epigenetic modulation to influence T-cell trafficking fates. Here, we dissect how reprogramming core metabolic networks calibrates trafficking cascades and highlight therapeutic strategies targeting key nodes to correct pathological migration in autoimmunity, cancer, and transplantation.

## 1. Introduction

T-cell trafficking is a multistep process by which T cells exit the bloodstream and enter target tissues, including secondary lymphoid organs and peripheral non-lymphoid tissues. This process is mediated by a series of receptor–ligand interactions and can be divided stepwise into: (i) chemokine receptor expression and chemotaxis; (ii) rolling and endothelial interactions; (iii) firm adhesion and integrin activation; (iv) transendothelial migration and interstitial motility; and (v) tissue entry and retention. Long-term tissue residency is regarded as a downstream consequence of trafficking rather than a trafficking step per se. T-cell differentiation, proliferation, survival, and effector molecule secretion are not encompassed by this definition unless they directly influence one of the aforementioned migratory steps. Under physiological conditions, trafficking enables distinct T-cell subsets to localize to sites of infection, tumors, or inflammation, thereby achieving immune surveillance and tissue homeostasis. Dysregulation of any trafficking step, whether arising from intrinsic cellular defects or microenvironmental perturbations, can lead to immune imbalance and tissue pathology [1].

Recent studies have shown that metabolic reprogramming can influence T-cell trafficking at multiple steps. In several experimental models, cytoskeletal remodeling and chemotactic migration depend on glycolysis for rapid energy production; the glycosylation modification and membrane localization of homing receptors are directly regulated by metabolic intermediates; and lipid metabolites participate in pseudopod formation and transendothelial migration [2,3,4,5]. These observations suggest a complex interplay between metabolic state and trafficking capacity, which is closely associated with infection immunity, tumor immunity, and autoimmunity [6,7,8]. Therefore, summarizing the dynamics of key enzymes and metabolites that regulate T-cell tissue tropism provides a direction for developing innovative immunotherapeutic strategies through modulation of metabolic pathways.

This review summarizes current evidence on how metabolic reprogramming regulates T-cell trafficking steps, including chemotaxis, rolling adhesion, firm adhesion, transendothelial migration, and interstitial migration For each type of metabolic reprogramming, we organize the discussion according to these trafficking steps and, where possible, distinguish direct evidence derived from migration assays or in vivo trafficking models from indirect evidence based on receptor expression, metabolic enzyme activity, or cell viability. We further discuss potential therapeutic strategies aimed at modulating T-cell trafficking through metabolic pathways, while highlighting current limitations in tissue selectivity and translational evidence.

## 2. Overview of T-Cell Trafficking

### 2.1. T-Cell Trafficking

T-cell trafficking refers to the process by which cells are directed to migrate to specific tissues, driven by a multistep molecular cascade initiated by receptor–ligand recognition on the vascular endothelium of target tissues [9]. This cascade begins with selectin-mediated rolling along the vessel wall. Subsequently, endothelial chemokines activate cell surface receptors, triggering conformational changes in integrins that mediate firm adhesion. The final step in leaving the blood is diapedesis, the passage of cells through the endothelial layer. The key molecule mediating this transendothelial migration is Platelet Endothelial Cell Adhesion Molecule-1 (PECAM-1)/Cluster of Differentiation 31 (CD31). PECAM-1 is expressed on leukocytes, platelets and endothelial cells, and is enriched at endothelial cell junctions. Homophilic PECAM-1–PECAM-1 interactions between the migrating cell and endothelial cells guide the cell through the endothelial barrier. After crossing, the cell migrates within the interstitial tissue and may establish early tissue residence. Under physiological conditions, trafficking is essential for immune defense and maintenance of tissue homeostasis [10,11]. However, dysregulated trafficking is associated with the pathogenesis of various diseases [12,13,14].

### 2.2. Trafficking of T-Cell Subsets

**Figure 1:**
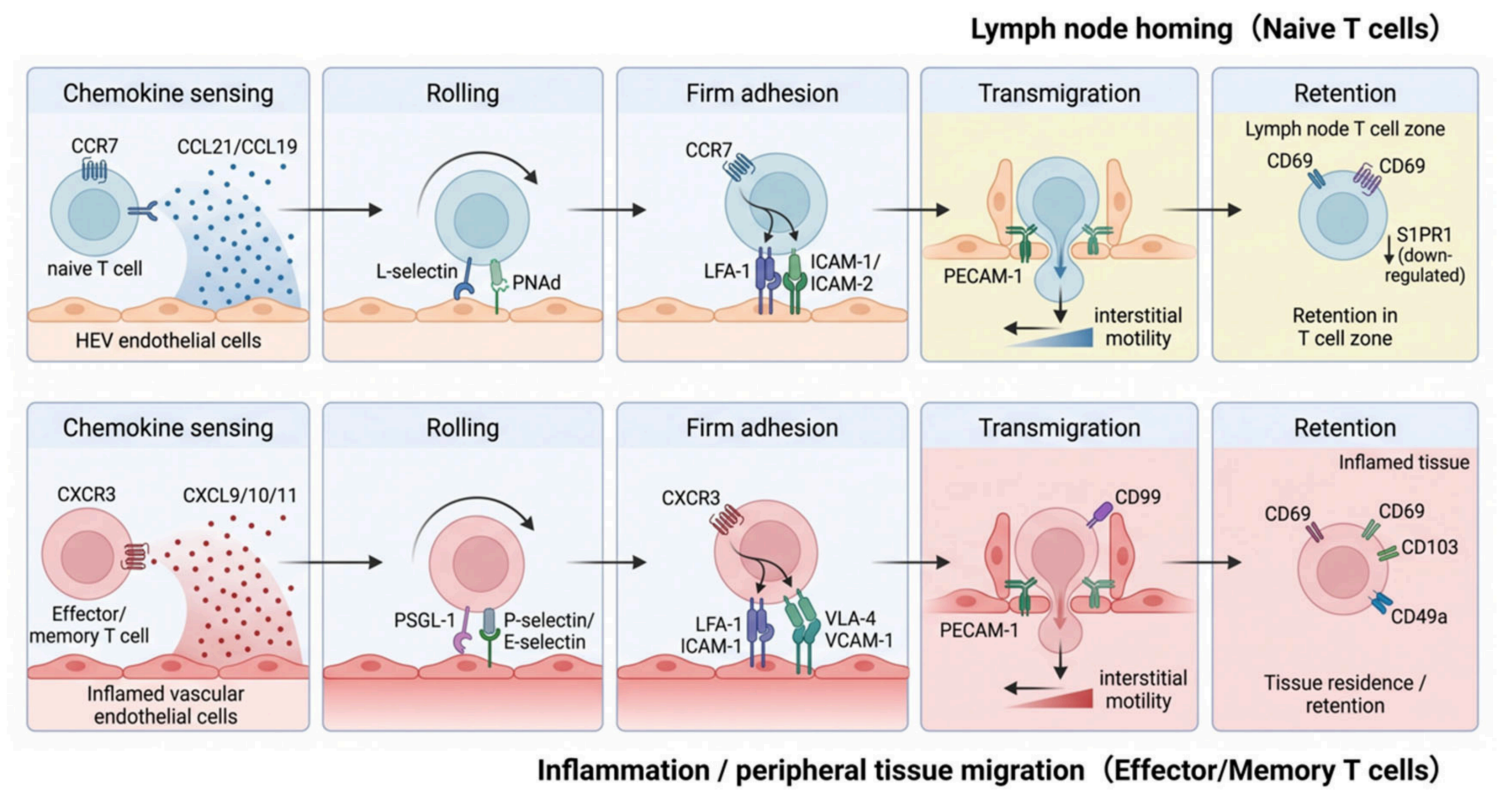
T-cell trafficking to lymphoid organs and inflamed tissues. T-cell trafficking is broadly divided into lymphoid organ homing and migration to peripheral or inflamed tissues. The figure depicts the multistep cascade by which T cells cross the vascular endothelium to enter specific tissues in these two canonical settings. Top, Naive T-cell homing to lymph nodes. Chemokine sensing is mediated by CCR7, which recognizes CCL21 and CCL19 displayed on high endothelial venules (HEVs). L-selectin binding to peripheral node addressin (PNAd) then mediates rolling; integrin LFA-1 binding to endothelial ICAM-1 and ICAM-2 mediates firm adhesion; and transendothelial migration depends on PECAM-1. Naive T cells ultimately enter the lymph node T-cell zone, where they upregulate CD69 and downregulate sphingosine-1-phosphate receptor 1 (S1PR1) to promote retention, with low interstitial motility. Bottom image: Effector/memory T-cell migration to inflamed tissues. Chemokine sensing is mediated by CXCR3, recognizing the inflammatory endothelial chemokines CXCL9, CXCL10 and CXCL11. Rolling depends on P-selectin glycoprotein ligand 1 (PSGL-1) binding to P-selectin and E-selectin. Firm adhesion is cooperatively mediated by LFA-1–ICAM-1 and VLA-4–VCAM-1 interactions. Transendothelial migration requires PECAM-1 and CD99, and is accompanied by markedly enhanced interstitial motility. Finally, upregulation of tissue-residency molecules such as CD69, CD103 and CD49a promotes T-cell retention and local effector function within inflammatory lesions.

T-cell heterogeneity determines the diversity of their migratory behavior. Different subsets enter specific tissues through distinct combinations of adhesion molecules, chemokine receptors and integrins. The trafficking process encompasses rolling, firm adhesion, diapedesis and interstitial migration, and some subsets subsequently establish early tissue residence. Long-term retention and effector functions are not part of the trafficking steps, but can influence their tissue distribution. The sequential steps of this trafficking process are schematically illustrated in Figure 1.

#### 2.2.1. Naïve T Cells

Naïve T cells, as antigen-inexperienced mature lymphocytes, continuously home to secondary lymphoid organs to perform immune surveillance. Their directional migration relies on high endothelial venules (HEVs): first, L-selectin on the T-cell surface binds to a peripheral node addressin (PNAd) expressed on HEVs, mediating rolling adhesion within the bloodstream. This is followed by the constitutive secretion of chemokines C-C motif chemokine ligand 19 (CCL19) and C-C motif chemokine ligand 21 (CCL21) from HEVs, which activate the C-C Chemokine Receptor Type 7 (CCR7) receptor on T cells. This triggering of G protein signaling induces a conformational change in integrin Lymphocyte Function-Associated Antigen 1 (LFA-1), enabling its high-affinity binding to endothelial Intercellular Adhesion Molecule-1/2 (ICAM-1/2) and resulting in firm adhesion. Furthermore, tissue-specific integrins regulate the targeting of this migration [15,16,17]. Under homeostatic conditions, naïve T cells enter lymph nodes via HEVs through the bloodstream, patrolling between secondary lymphoid organs. However, during inflammation or infection, HEV injury leads to the downregulation of PNAd and CCL21. Consequently, the cells switch to an L-selectin-independent compensatory pathway: they migrate into tissue lymphatic vessels via Vascular Endothelial Growth Factor C (VEGF-C)/Vascular Endothelial Growth Factor Receptor 3 (VEGFR3)-mediated lymphangiogenesis, and rely on the upregulation of Lymphocyte Peyer’s patch adhesion molecule 1 α4β7 (Lymphocyte Peyer’s patch adhesion molecule 1, LPAM-1) integrin to bind to Mucosal Vascular Addressin Cell Adhesion Molecule 1 (MAdCAM-1) on the lymphatic endothelium, ultimately gaining access to the draining lymph nodes [18]. This trafficking process serves as the cornerstone for initiating adaptive immunity. On the one hand, it ensures efficient antigen surveillance within secondary lymphoid organs (SLOs), thereby supporting antigen-specific responses. On the other hand, during chronic infections, it maintains the supply of naïve T cells to the lymph nodes via compensatory pathways, thereby promoting the expansion of effector T cells to sustain immune defense [18,19,20].

#### 2.2.2. Effector T Cells

Effector T cells migrate to peripheral sites of infection or inflammation. The Effector Cluster of Differentiation 8-positive (CD8^+^) T cells highly express inflammatory chemokine receptors, such as C-X-C motif chemokine receptor 3 (CXCR3), C-C motif chemokine receptor 4 (CCR4), C-C motif chemokine receptor 6 (CCR6), C-C motif chemokine receptor 9 (CCR9) and C-C motif chemokine receptor 10 (CCR10), enabling them to respond to ligands such as C-X-C motif chemokine ligand 9 (CXCL9), C-X-C motif chemokine ligand 10 (CXCL10), C-X-C motif chemokine ligand 11 (CXCL11) or C-C motif chemokine ligand 25 (CCL25) [21]. The C-X-C motif chemokine ligand 12(CXCL12)-C-X-C Chemokine Receptor Type 4(CXCR4) axis constitutes a critical pathway for bone marrow trafficking. However, Interleukin-2 (IL-2)-mediated ex vivo expansion or persistent Chimeric Antigen Receptor (CAR) signaling can lead to excessive mechanistic target of rapamycin complex 1 (mTORC1) activation. This, in turn, suppresses the transcription factor Forkhead Box Protein O1/3 (FOXO1/3), resulting in the downregulation of CXCR4 and thereby impairing the cell’s responsiveness to the stromal chemokine CXCL12 [22]. Integrins mediate tissue-specific anchoring. The α4β7 integrin binds to the gut mucosal addressin MAdCAM-1, which drives trafficking to the intestines [23,24,25]; meanwhile, Very Late Antigen-4 (VLA-4) and Very Late Antigen-1 (VLA-1) regulate interstitial migration and tissue retention by recognizing fibronectin and collagen, respectively [26,27,28]. The selectin pathway mediates initial endothelial adhesion. Following systemic viral infection, activated effector CD8^+^ T cells broadly express P-/E-selectin ligands [29], Through endogenous fucosylation, they dynamically modify Cluster of Differentiation 43 (CD43) to generate sialyl Lewis X (sLeX) structures, which bind to E-selectin on bone marrow endothelium to initiate rolling adhesion, However, enforced exogenous fucosylation has failed to enhance the bone marrow trafficking efficiency of CAR-T cells. More notably, the therapeutic advantages demonstrated by natural cytokines like IL-2, which act through endogenous regulation of trafficking molecules, highlight a more clinically viable optimization pathway compared to artificial enforced engineering [26].

#### 2.2.3. Memory T Cells

Memory T cells, the long-lived guardians of immunity, are classified into central memory T-cell (TCM), effector memory T-cell (TEM), and tissue-resident memory T-cell (TRM) subsets. The trafficking patterns are different for TCM trafficking to lymph nodes as it initiates with Cluster of Differentiation 62L (CD62L)-mediated rolling on PNAd, progresses through CCR7-dependent sensing of CCL19/21 gradients, and culminates in LFA-1 activation, leading to firm adhesion and diapedesis into the paracortex [30,31]. The trafficking of TEM to peripheral inflammatory sites or specific tissues is more diverse. Their tissue-specific trafficking is mediated by receptors such as the α4β7 integrin binding to MAdCAM-1 expressed on gut vascular endothelium [32,33], as well as CCR9 responding to the chemokine CCL25 secreted by small intestinal epithelium. Meanwhile, their recruitment to inflammatory sites involves inflammatory signals (e.g., Interleukin-15, IL-15) inducing the expression of core 2 O-glycans (e.g., P-selectin glycoprotein ligand-1, PSGL-1) that serve as ligands for P- and E-selectin to mediate rolling adhesion [34,35]; this is guided by chemokine receptors (e.g., C-X-C Chemokine Receptor Type 3, CXCR3 responding to CXCL9/CXCL10 and CCR5 responding to C-C Motif Chemokine Ligand 3/4/5, CCL3/4/5), directing transendothelial migration to the site of inflammation [36,37,38]. The establishment of TRM is a continuum comprising initial recruitment and subsequent long-term retention. Tissue-specific trafficking receptors, including C-X-C Chemokine Receptor Type 6 (CXCR6)/C-X-C motif chemokine ligand 16 (CXCL16) for the liver [39,40] and Somatostatin Receptor Type 2 (SSTR2)/somatostatin for the gut [41], mediate initial recruitment. In contrast to their recruitment, long-term residency is maintained via Cluster of Differentiation 69 (CD69)-dependent sphingosine-1-phosphate receptor 1 (S1PR1) internalization to block egress [42], Cluster of Differentiation 103 (CD103)–E-cadherin interactions for epithelial retention [40,43], and local survival signals from Transforming Growth Factor Beta (TGF-β), IL-15, and retinoic acid that sustain the TRM transcriptional program [44].

TCM subsets achieve functional complementarity through distinct trafficking mechanisms: serving as the lymph node-trafficking “memory hub,” TCMs rapidly expand upon antigen re-encounter to provide systemic immune protection and antitumor responses [45]; TEM cells act as a peripheral “rapid-response force” that is recruited to infection sites in an inflammation-independent manner to exert immediate effector functions. Specific activated subsets, such as CD103^+^β7^+^CD38^+^ CD8^+^ TEM cells, serve as non-invasive diagnostic biomarkers for conditions like celiac disease [33], yet under chronic inflammatory conditions, they can transform into drivers of pathological damage [32,37]; In contrast, TRM cells function as “lifelong sentinels” at barrier tissues, mediating near-instant immune responses [46]. Certain TRM subsets (e.g., CD103^−^ TRM in the liver) exhibit cross-tissue plasticity and can remobilize to establish an “immune once, defend multiple sites” network [42,47], thereby providing a fundamental basis for the design of targeted mucosal vaccines [46].

#### 2.2.4. Regulatory T Cell (Treg Cells)

Treg cells, a specialized subset of CD4^+^ T cells characterized by the expression of the transcription factor Forkhead box P3 (Foxp3), traffick to specific tissues where they maintain immune tolerance, suppress inflammation, and regulate tissue repair. They achieve this primarily by inhibiting the activation and proliferation of effector T cells through either direct cell contact or the secretion of inhibitory factors. Naive Tregs rely on the CCR7 receptor to recognize CCL19/CCL21 chemokines secreted by lymph node endothelial cells and utilize CD62L to mediate rolling adhesion, thereby facilitating their trafficking to the T-cell zone. Following antigen activation, Tregs downregulate CCR7 and CD62L while upregulating C-X-C Chemokine Receptor Type 5 (CXCR5), enabling their migration into germinal centers to participate in the regulation of immune responses [48,49,50]. The trafficking of lymphocytes to the intestine involves multi-layered pathways: the α4β7 integrin binds to MAdCAM-1 to mediate endothelial adhesion; the CCR9-CCL25 chemokine axis directs their migration to the small intestine; and the G Protein-Coupled Receptor 15 (GPR15)–G Protein-Coupled Receptor 15 Ligand (GPR15L) axis, under the regulation of the Aryl hydrocarbon receptor (AhR)-Foxp3-retinoic acid receptor (RAR)-related orphan receptor gamma t (RORγt) transcriptional network, specifically mediates trafficking to the colon [51,52,53]. Tumor-associated Treg cells are recruited to the tumor site via the CCR4–C-C motif chemokine ligand 22 (CCL22) axis, while their migration to inflammatory sites relies on the CXCR3–CXCL9/10 chemokine gradient. Upon activation, Tregs upregulate the expression of integrin alpha-E beta-7 (αEβ7), which facilitates their long-term retention within inflamed tissues by binding to epithelial E-cadherin [50,54]. Recent advances in engineering strategies have significantly enhanced the tissue-specific enrichment of Treg cells through the overexpression of trafficking receptors, such as C-X3-C Motif Chemokine Receptor 1 (CX3CR1) for plaque targeting and GPR15/CCR9 for gut-specific migration [53,55]. Consequently, the bidirectional immunomodulatory function mediated by Treg trafficking represents a promising foundation for precision immunotherapy.

## 3. The Impact of Metabolic Reprogramming on T-Cell Trafficking

Metabolic reprogramming refers to an adaptive mechanism whereby cells dynamically adjust their metabolic pathways in response to physiological or pathological conditions, such as environmental stress or aberrant proliferative demands. Its hallmark is the rewiring of metabolic flux to support cell survival and function under changing conditions [56]. Based on the metabolic pathways involved or underlying regulatory mechanisms, metabolic reprogramming can be categorized into several types, including glucose metabolism reprogramming, amino acid metabolism reprogramming, lipid metabolism reprogramming, and mitochondrial metabolic reprogramming. Key triggers driving these alterations encompass oncogene activation, microenvironmental stress, epigenetic modifications, and immune metabolic switching [57,58]. The dynamic plasticity of metabolic reprogramming orchestrates the entire T-cell trafficking cascade through differential allocation of energy substrates, stage-specific generation of metabolic intermediates, and coordinated activation of chemokine signaling pathways. These mechanisms operate in a highly stage-dependent manner to fine-tune immune cell trafficking and responses (as summarized in Table 1).

### 3.1. Glycolytic Metabolic Reprogramming

Glycolytic metabolic reprogramming denotes the process by which cells dynamically adapt their glucose metabolic pathways in response to environmental cues. The key pathways involved include glycolysis, the pentose phosphate pathway, the hexosamine biosynthesis pathway, and glycogen synthesis [59,60,61,62]. Recent studies have revealed that its regulatory role in T-cell trafficking extends beyond mere energy provision. Instead, glycolytic metabolic reprogramming orchestrates molecular events throughout the trafficking cascade via metabolic intermediates such as nucleotide precursors, receptor glycosylation modifications, and intricate interactions with metabolic signaling pathways.

#### 3.1.1. Chemokine Receptor Expression and Chemotaxis

Direct experimental evidence supports a role for glycolysis in T-cell chemotaxis. Kishore et al. [63] demonstrated in in vitro chemotaxis assays that pro-migratory stimuli induce glucokinase expression and initiate glycolysis through the phosphatidylinositol 3-kinase (PI3K)–mechanistic target of the rapamycin complex 2 (mTORC2) pathway, and that inhibition of glycolysis with 2-deoxyglucose (2-DG) significantly impairs the chemotactic capacity of Treg cells. The same study also showed through adoptive transfer experiments that Treg cells with glucokinase-dependent glycolysis deficiency exhibited markedly reduced migration to skin grafts, thereby confirming at the in vivo level the necessity of glycolysis for Treg chemotactic migration. Similarly, Liu et al. [64] demonstrated through Transwell chemotaxis assays and in vivo adoptive transfer experiments that CD31 Y686F mutation or CD31 knockout significantly impairs the migratory capacity of Treg cells to secondary lymphoid tissues; mechanistically, the Y686F mutation disrupts the glycolytic and fructose metabolic switch, and extracellular acidification rate (ECAR) assays directly confirmed that Y686F mutation and CD31 knockout suppress glycolytic activity in Tregs. Another study [65] also showed that CD31-mediated signals attenuate T-cell chemokinesis both in vitro and in vivo, with this effect acting selectively on activated/memory T lymphocytes. Furthermore, lactate, the end product of glycolysis, can negatively regulate T-cell motility in a feedback manner: Haas et al. [66] found that lactate inhibits CD4^+^ T-cell motility by interfering with glycolytic signaling downstream of CXCR3, suggesting bidirectional regulation between glycolytic activity and chemotactic responses. Moreover, functional Transwell migration assays have demonstrated that double-negative T cells, which suppress glycolysis, exhibit enhanced migration toward CCR7 and CXCR5 ligands while their migration toward CXCR3 ligands is reduced, directly linking glycolytic modulation to altered chemotactic capacity [67].

At the level of receptor expression and transcriptional regulation, multiple studies have provided indirect evidence. In diabetic mice, CD8^+^ T cells exhibit impaired glycolysis, accompanied by reduced CCR7 expression and diminished chemotactic responses to CCL21, and this defect can be partially restored by anti-interferon alpha/beta receptor subunit 1 (IFNAR1) treatment [68]. Overexpression of carbohydrate kinase-like (CARKL), a key enzyme in the pentose phosphate pathway, impairs CXCR3 expression in T cells and attenuates their migratory responses to cognate chemokines [69]. Runt-related transcription factor 2 (RUNX2) overexpression enhances the glycolytic program and reinforces CXCR4 signaling, thereby promoting chemotactic migration of T-cell acute lymphoblastic leukemia (T-ALL) cells toward CXCL12 [70]. Furthermore, CD31, upon phosphorylation of its immunoreceptor tyrosine-based inhibitory motif (ITIM) motif, recruits Src homology 2 domain-containing protein tyrosine phosphatase 2 (SHP2) to regulate the T-cell activation threshold; Tregs bearing the CD31 Y663F mutation exhibit reduced glucose uptake together with enhanced 6-phosphofructo-2-kinase/fructose-2,6-biphosphatase 3 (PFKFB3)-regulated fructose utilization, a glucose-to-fructose metabolic switch that further suggests a potential role for the glycolysis–fructose metabolism balance in T-cell migration. Collectively, these results indicate that the glycolytic state can influence the migratory direction of T cells by altering chemokine receptor expression patterns and metabolic signaling.

#### 3.1.2. Rolling and Endothelial Interactions

There is relatively direct experimental evidence that glycolytic branch pathways regulate the rolling step. Synthesis of surface selectin ligands such as sialyl Lewis X (sLeX) depends on the hexosamine biosynthetic pathway (HBP). Blockade of this pathway with 4-fluoro-N-acetylglucosamine (4-F-GlcNAc) significantly downregulates E-selectin ligand expression on the T-cell surface and inhibits initial tethering to endothelial cells [71]. More directly, treatment of human CLA+ T cells with 4-F-GlcNAc, which inhibits poly-N-acetyllactosamine elongation downstream of the HBP, markedly reduced their rolling on E-, P-, and L-selectin under physiological shear stress; this effect was accompanied by decreased HECA-452 reactivity and direct incorporation of 4-F-GlcNAc into PSGL-1 O-glycans [72]. These findings demonstrate that branch products of glucose metabolism participate in selectin ligand glycosylation in T cells themselves, thereby modulating rolling adhesion. Overall, compared with chemotaxis, primary studies directly targeting T-cell glycolysis or the HBP in the rolling step remain relatively scarce, although existing evidence supports a critical role for the HBP in this process.

#### 3.1.3. Firm Adhesion and Integrin Activation

The role of glycolysis in integrin-mediated firm adhesion is supported by relatively direct experimental evidence. Kamnev et al. [73] demonstrated through cell spreading and adhesion assays that inhibition of glycolysis impairs lamellipodial filamentous actin (F-actin) remodeling, chemokine-driven motility, and LFA-1-dependent adhesion in human CD8^+^T cells, whereas inhibition of mitochondrial oxidative phosphorylation (OXPHOS) exerts a milder effect on F-actin, primarily reducing cell elongation during confined migration. This indicates that glycolysis provides important support for integrin-mediated firm adhesion. Consistent with this, the study by Kishore et al. [74] showed that stimulation of pro-migratory receptors, including LFA-1, can initiate glycolysis in Treg cells, suggesting bidirectional coupling between integrin signaling and glycolysis. Guak and Krawczyk [75] further discussed this relationship from the perspective of integrating metabolism with integrin function. Collectively, glycolysis participates in the regulation of the firm adhesion step by supporting actin remodeling and the formation of adhesion structures.

#### 3.1.4. Transendothelial Migration and Interstitial Motility

The role of glycolysis in transendothelial migration and interstitial motility exhibits stage-dependent and bidirectional characteristics. In transendothelial migration assays, Kishore et al. [74] demonstrated that inhibition of glycolysis did not significantly affect Treg cell crossing of the endothelial monolayer, but markedly impaired their chemotaxis, suggesting that the contribution of glycolysis at this stage may be primarily concentrated in upstream chemotactic steps rather than in the transendothelial process itself. In terms of interstitial motility, Haas et al. showed that lactate inhibits CD4^+^T-cell motility by interfering with the CXCR3–glycolysis axis through solute carrier family 5 member 12 (SLC5A12); concurrently, lactate also inhibits CD8^+^T-cell motility in a glycolysis-independent manner via solute carrier family 16 member 1 (SLC16A1) [66]. These results indicate that lactate, the terminal product of glycolysis, can negatively regulate interstitial motility of T cells. Some clinically relevant observations report that adipose tissue T cells from individuals with HIV, despite a high expression of CXCR4 and CCR7, may exhibit reduced migratory efficiency due to impaired glucose tolerance [76], providing indirect evidence that metabolic dysfunction can influence T-cell migration. Collectively, the direct contribution of glycolysis to transendothelial migration appears limited, whereas in interstitial motility, glycolysis may both promote movement by supporting microfilament remodeling and inhibit movement through lactate accumulation, thereby displaying bidirectional regulatory features.

**Figure 2:**
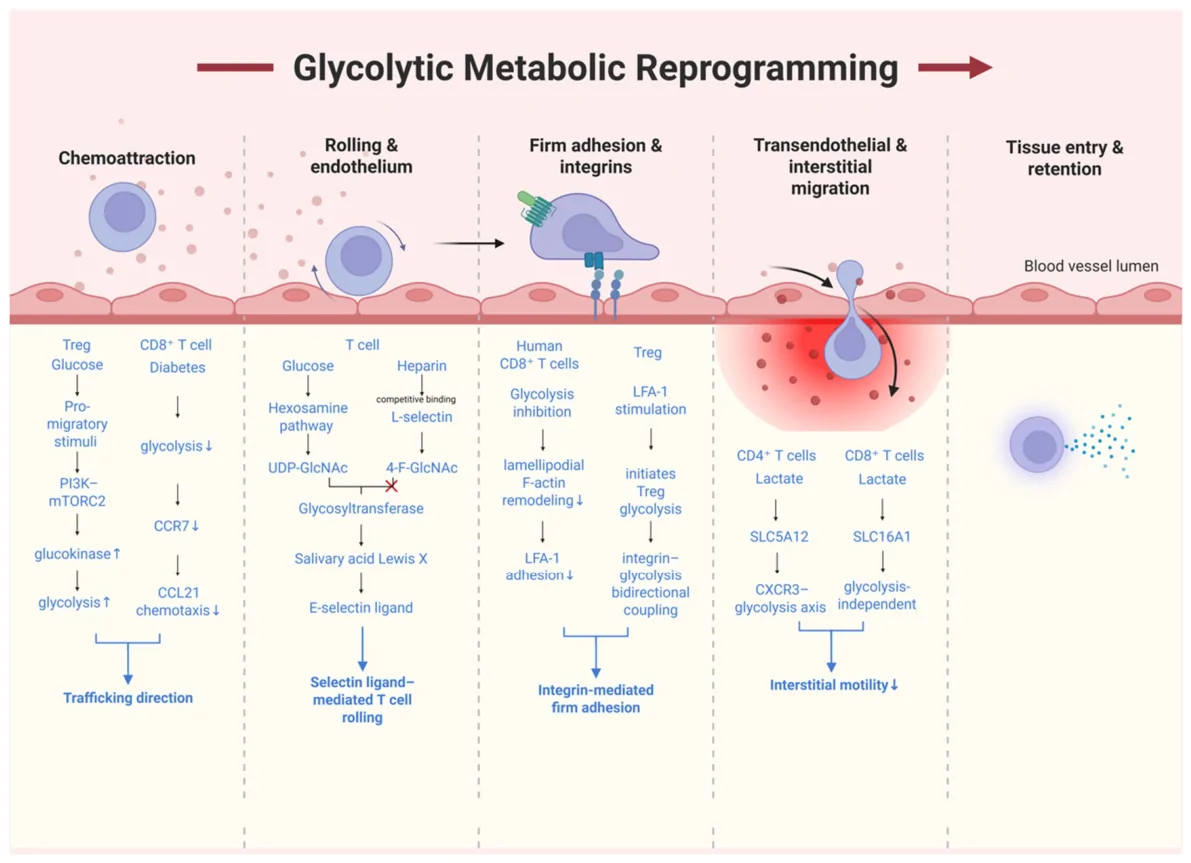
Schematic diagram of glucose metabolic reprogramming of regulatory nodes in T-cell trafficking. This schematic depicts the stepwise regulatory mechanisms by which glycolytic metabolic reprogramming governs the entire process of T-cell transendothelial trafficking. The migratory process is divided into four stages, each exemplified by distinct T-cell subsets and their corresponding regulatory pathways. In the chemotaxis stage, regulatory T cells and CD8^+^T cells under diabetic conditions are used as examples: the former upregulate glycolysis through the PI3K–mTORC2 pathway to enhance chemotactic capacity, while the latter exhibit impaired glycolysis that downregulates CCR7 and attenuates chemotactic responses, together regulating the direction of cell trafficking. In the rolling adhesion stage, effector T cells are exemplified: glucose generates E-selectin ligands via the hexosamine biosynthetic pathway to mediate initial rolling, and exogenous heparin competitively inhibits this process without representing cell-intrinsic metabolic regulation. In the firm adhesion stage, human CD8^+^T cells and regulatory T cells are exemplified: inhibition of glycolysis in the former impairs LFA-1-dependent adhesion, while in the latter, there is bidirectional coupling between LFA-1 signaling and glycolysis. In the transendothelial and interstitial motility stage, CD4^+^and CD8^+^T cells are exemplified: lactate interferes with the CXCR3–glycolysis axis via SLC5A12 to inhibit interstitial motility of CD4^+^T cells, and inhibits interstitial motility of CD8^+^T cells via SLC16A1 in a glycolysis-independent manner; moreover, glycolysis has no significant direct effect on transendothelial migration per se. Arrows indicate the direction of metabolic pathways/regulatory cascades and denote promotion, activation, or downstream effects; the symbol ↓ indicates downregulation or decreased activity. Red × indicates inhibition/blockade of the indicated step.

Collectively, the regulation of T-cell trafficking by glycolysis is supported by relatively clear direct evidence in the chemotaxis, firm adhesion, and rolling steps, whereas evidence in interstitial motility remains insufficient or bidirectional. Existing studies suggest that the role of glycolysis extends beyond energy supply to involve receptor expression, selectin ligand glycosylation, actin remodeling, and metabolite signaling feedback; nevertheless, the specific contributions and regulatory mechanisms at different steps still require further discrimination and validation. Existing studies suggest that the role of glycolysis extends beyond energy supply to involve receptor expression, selectin ligand glycosylation, actin remodeling, and metabolite signaling feedback; nevertheless, the specific contributions and regulatory mechanisms at different steps still require further discrimination and validation. The stage-specific roles and regulatory mechanisms of glycolytic metabolic reprogramming in T-cell trafficking are summarized in Figure 2.

### 3.2. Amino Acid Metabolic Reprogramming

Amino acid metabolic reprogramming is defined as a core adaptive mechanism whereby cells dynamically modulate the uptake, catabolism, and utilization of amino acid-derived metabolites to sustain energy production, biosynthetic demands, and signaling transduction under specific physiological or pathological conditions. This process encompasses major pathways such as glutaminolysis, aspartate metabolism, arginine metabolism, and tryptophan metabolism [77,78,79,80]. Collectively, these pathways shape the metabolic phenotype and functional plasticity of the cell by regulating key signaling hubs, including mTORC1 and the AhR.

**Table 1:** Summary of evidence for metabolic regulation of T-cell trafficking.

Metabolic Pathway	Trafficking Stage	Cell Type	Metabolic Perturbation	Experimental Model	Readout	Evidence Level	Reference
Glycolytic Metabolic Reprogramming	Chemokine receptor expression and chemotaxis; Transendothelial migration	Treg	mTORC2-PI3K-Akt axis; glucokinase	In vitro cell culture; Transwell chemotaxis/transendothelial	In vitro migration; Treg infiltrating cell count in inflamed site	Direct	[63]
	Chemokine receptor expression and chemotaxis	Treg	CD31-SHP2; PFKFB3;	Transwell chemotaxis	In vitro chemotaxis migration efficiency; ECAR	Direct	[64]
	Chemokine receptor expression and chemotaxis	CD4^+^, CD8^+^ T cells	Slc5a12, Slc16a1	Transwell chemotaxis and transendothelial migration; Seahorse extracellular flux assay	In vitro chemotactic migration rate; ECAR; glucose uptake; count of intra-peritoneal T-cell infiltration	Direct	[66]
	Chemokine receptor expression and chemotaxis	DN Treg	mTOR-HIF-1α; GLUT1/GLUT3	Transwell chemotaxis assay; Transwell separated indirect co-culture system; flow cytometry for chemokine receptors	In vitro receptors: CXCR3, CCR5, integrin α4β7 downregulated; CCR7, CXCR5 upregulated. In vitro migration: reduced chemotaxis to CXCL10, CCL3; no significant change for CXCL9	Direct	[67]
	Chemokine receptor expression and chemotaxis	CD8^+^ T cells	IFN-α/β; IFNAR1	Transwell CCL21 chemotaxis assay	In vitro CCL21-dependent chemotactic efficiency of CD8^+^ T cells; CCR7, CD127	Direct	[68]
	Chemokine receptor expression and chemotaxis	CD4^+^ T cells	CARKL overexpression	Transwell chemotaxis; qPCR for chemokine receptor transcripts	In vitro chemotactic efficiency towards CXCL10; CXCR3, CCR4, CCR7	Direct	[69]
	Chemokine receptor expression and chemotaxis; Firm adhesion and integrin activation	T-ALL cells	RUNX2 overexpression	Transwell CXCL12 chemotaxis	CXCR4 expression; CXCL12-directed chemotactic migration rate	Direct	[70]
	Rolling and endothelial interactions; Firm adhesion and integrin activation	effector T cells; NK cells	4-F-GlcNAc; fucosyltransferase 4/7; PSGL-1	Parallel-plate flow-chamber rolling assay; Flow-cytometric quantification of E-selectin-ligand-positive T/NK cells within skin-draining inguinal lymph nodes.	E-/P-selectin ligand expression; leukocyte rolling capacity on selectins; cell adhesion; PSGL-1	Direct	[71]
	Rolling and endothelial interactions	Effector T cells	4-F-GlcNAc; L-selectin; P-selectin	Parallel-plate flow-chamber assay: quantitate lymphocyte tethering and rolling on recombinant E-/P-/L-selectin-Ig under physiological shear stress, recapitulating the initial adhesion step of leukocyte extravasation.	Effector T cells show 5-fold elevated E-selectin ligand and enhanced in vitro rolling on E-selectin	Direct	[72]
	Chemokine receptor expression and chemotaxis; Firm adhesion and integrin activation	CD8^+^ T cells	OXPHOS; actin related protein 2/3 complex	In vitro high-content cell imaging; under-agarose confined-migration assay; Ibidi chemotaxis chamber with CXCL12 gradient	Chemotactic migration speed; track parameters; cell-adhesion capacity; LFA-1 activation status	Direct	[73]
	Chemokine receptor expression and chemotaxis; Firm adhesion and integrin activation	T cells	Dynamin-related protein 1; mitochondrial Rho GTPase 1; HIF-1α; mTOR-AMPK	Transwell chemotaxis; parallel-plate flow chamber; transendothelial migration; Seahorse metabolic profiling	CCR7, CXCR4; in vitro chemotactic index; in vivo immune-cell tissue infiltration	Direct	[75]
	Chemokine receptor expression and chemotaxis; Transendothelial migration and interstitial motility	CD4^+^, CD8^+^ memory T cells	CXCR1; CXCR2; CXCR4; CCL5; CD69; CX_3_CR1; GPR56; CD57	Flow cytometry of PBMC and adipose-derived T cells	Frequency of CD4^+^/CD8^+^ memory T-cell subsets in adipose tissue; expression level of CD69, CD57, CX_3_CR1, GPR56	Indirect	[76]
Amino Acid Metabolic Reprogramming	Chemokine receptor expression and chemotaxis; Rolling and endothelial interactions	CD8^+^ T cells	Vacuolar protein sorting 34	Flow-cytometry-based autophagy-flux measurement; DIA quantitative proteomics; Seahorse extracellular flux assay	In vivo CD8^+^ T-cell differentiation phenotypes; CD62L	Indirect	[81]
	Chemokine receptor expression and chemotaxis	CD4^+^ T cells	Indoleamine 2,3-dioxygenase 1/2; Gpr15; AhR	HH7-2 TCR adoptive transfer; in vitro AhR ligand screening in CD4^+^ T cells	Count of GPR15^+^FOXP3^+^ Treg in colonic lamina propria; Gpr15	Indirect	[82]
	Chemokine receptor expression and chemotaxis	CTL	DAPK1; mTORC1	Ex vivo CD8^+^ T-cell activation culture; qPCR for homing-receptor transcripts; flow cytometry for phosphorylated-S6	Expression level of CD62L, CCR7, CXCR3, KLF2; CD8^+^ T-cell infiltration count	Indirect	[83]
	Chemokine receptor expression and chemotaxis	CD4^+^ T cells	Not assessed	HLA-class-II tetramer plus 24-color spectral flow cytometry; longitudinal PBMC specimen analysis	Circulating frequency of citrulline-specific CD4^+^ T cells; PD-1; CD95	Indirect	[84]
	Chemokine receptor expression and chemotaxis	CD4^+^ Th17, CD8^+^ Tc17 cells	Fumaric acid esters; Mycophenolate mofetil; α-ketoglutarate-dependent demethylases	Primary T-cell Th17/Tc17 polarization cultures; Illumina EPIC 850K DNA-methylation BeadChip; flow cytometry for chemokine-receptor profiling	MIR-21; SMAD7; CCR6; Th17/Tc17 subset proportions	Indirect	[85]
	Rolling and endothelial interactions	CD4^+^ Th, CD8^+^ Tc effector T cells	Dietary glutamine supplementation	qPCR for endothelial adhesion molecules; flow cytometry of lymphocytes from peripheral blood/mesenteric lymph nodes	PSGL-1; LFA-1; α4β7-integrin; CCR9; ICAM-1; E-selectin; P-selectin; mucosal CD4^+^/CD8^+^ T-cell infiltration	Indirect	[86]
	Rolling and endothelial interactions	CLA^+^ T cells	Spermine NONOate; E-selectin	Primary dermal microvascular endothelial cells/human umbilical vein endothelial cells endothelial co-culture; magnetic-activated cell-sorting of MDSCs; tumor-tissue immunofluorescence/flow cytometry	Percentage of E-selectin-positive tumor vessels; density of tumor-infiltrating CLA^+^ T cells	Indirect	[87]
	Rolling and endothelial interactions	CD4^+^, CD8 T cells	Glutamine; STAT3; CCL5	Ex vivo HGEC culture; qRT-PCR, Western blot, ELISA; Transwell chemotaxis co-culture system	ASCT2; CCL5; STAT3	Indirect	[88]
	Firm adhesion and integrin activation	Th17 cells; CD4^+^ T cells	Glutaminase; β1-integrin (CD29) -VCAM-1; Kv1.3	In vitro Th17 polarization culture	Glutaminase mRNA and protein; VAMP2/3/4, SNAP23; β1-integrin, Kv1.3 expression	Indirect	[89]
	Firm adhesion and integrin activation; Chemokine receptor expression and chemotaxis	T cells	Glutamate/α-amino-3-hydroxy-5-methyl-4-isoxazolepropionic acid; CD29, VLA-6; CXCR4	Ex vivo primary human T-cell culture; Boyden-chamber-based CXCL12 chemotaxis assay	Chemotactic migration efficiency towards CXCL12; CXCR4	Indirect	[90]
	Firm adhesion and integrin activation	Th2 cells	mTORC1	Single-cell metabolic assay; RNA-seq; flow cytometry	CCR8, ICAM1); Amphiregulin; in vivo numbers of adipose-tissue resident Th2 cells	Indirect	[91]
	Chemokine receptor expression and chemotaxis; Transendothelial migration and interstitial motility; Firm adhesion and integrin activation	CD8^+^ T cells	TCA; glutaminase; PDHA1/OGDH	Transwell transendothelial migration; in vivo CAR-T infiltration and antitumor functional assays	Cell migration speed; Transwell-based transmigration across TNF-activated HUVEC monolayers; proportion of CAR-T infiltrating tumor islets	Direct	[92]
Lipid Metabolic Reprogramming	Chemokine receptor expression and chemotaxis	CD4^+^ T cells	EPA/DHA; ω-3 PUFA; 12-HEPE, 9-HOTrE, 19,20-DiHDPA, AEA, C1P	Intra-peritoneal CXCL10-driven recruitment; Transwell transendothelial migration	In vitro transendothelial-migration efficiency; in vivo peritoneal T-cell recruitment; CXCR3	Direct	[93]
	Chemokine receptor expression and chemotaxis; Firm adhesion and integrin activation	CD4^+^ effector T cells	Pantethine treatment	Confocal microscopy for CXCR4/CXCR7 localization; Transwell chemotaxis and transendothelial-migration assay	Membrane localization and internalization of CXCR4/CXCR7; T-cell chemotaxis, transendothelial migration and adhesion	Direct	[94]
	Chemokine receptor expression and chemotaxis; Rolling and endothelial interactions	CD4^+^ Foxp3^+^ regulatory T cells	PPARδ; CPT1; mTOR	Treg adoptive transfer; ex vivo aortic-infiltration assay; Transwell chemotaxis	Lymph-node homing capacity of Treg; expression of CD62L, CCR7, S1PR1, KlF2; activity of mTORC1, mTORC2; migratory efficiency of Treg toward inflamed peritoneum and atherosclerotic lesions	Direct	[95]
	Chemokine receptor expression and chemotaxis; Rolling and endothelial interactions	CD4^+^/CD8^+^ T cells	Lung Kruppel-like factor	Flow-cytometric Fas/FasL measurement; ex vivo T-cell activation culture	L-selectin, CD44, CD69; abundance of SP-T cells in peripheral lymphoid organs and circulation	Indirect	[96]
	Chemokine receptor expression and chemotaxis	CD4^+^ T cells	KlF2; S1P_1_; CCR1/CCR5	Intrathymic fluorescein isothiocyanate labeling for thymic-egress tracing; adoptive-transfer in vivo homing assay; Transwell in vitro chemotaxis	mRNA and protein levels of S1P_1_, CCR1, CCR3, CCR5, CXCR1-3; thymic-egress efficiency; T-cell distribution in spleen, lymph nodes, liver and other organs; in vitro chemotactic response toward S1P and CCL11	Direct	[97]
	Chemokine receptor expression and chemotaxis; Rolling and endothelial interactions	CTL	Phosphoinositide-dependent kinase 1; PH-domain K465E knock-in mutation (abolishes PI(3,4,5)P_3_ binding)	qPCR for chemokine/homing receptors; in vivo adoptive-transfer homing assay; flow-cytometric phenotyping	Transcript and protein levels of KLF2, CD62L, CCR7, S1P_1_; T-cell proliferation, viability,	Indirect	[98]
	Chemokine receptor expression and chemotaxis	CD4^+^ T cells	IL-4Rα; retinoic-acid-receptor antagonist LE135 blockade	Ex vivo co-culture of MLN-DC with antigen-specific naive CD4^+^ T cells; flow cytometry for homing-receptor profiling; adoptive-transfer-based in vivo tissue distribution assay	Retinaldehyde dehydrogenase 2 (RALDH2); CCR9; α4β7; CD62L; CD44	Indirect	[99]
	Chemokine receptor expression and chemotaxis	T cells	PPAR-γ; RALDH-inhibitor	ALDEFLUOR-based RALDH enzymatic-activity assay; LC-MS; immunofluorescence co-localization on human gut-associated lymphoid tissue tissues	mRNA and protein abundance of RDH10, RALDH2, CRABP2, TGM2, CD1D; DC-mediated competence to imprint gut-homing programs in T cells	Indirect	[100]
	Chemokine receptor expression and chemotaxis	CD4^+^/CD8^+^ T cells	HFD-induced obesity	Transwell in vitro chemotaxis assay; hepatic immunofluorescence/immunohistochemistry	Number of hepatic-infiltrating CD4^+^/CD8^+^ T cells; in vitro lymphocyte chemotactic responsiveness towards CXCL12/CXCL13/CCL19/CCL21	Direct	[101]
	Rolling and endothelial interactions	CD4^+^ T cells	MβCD-mediated membrane-cholesterol depletion	Ex vivo T-cell culture; static adhesion assay; parallel-plate flow-chamber shear-flow rolling-adhesion assay	Static adhesion percentage to HA; rolling frequency and rolling velocity under physiological shear stress; rolling-adhesion efficiency on endothelial monolayer	Indirect	[102]
	Rolling and endothelial interactions; Firm adhesion and integrin activation	T cells	Duodenal olive oil/octanoic acid administration	Adoptive transfer of CFSE-labeled T lymphocytes; intravital fluorescence microscopy of Peyer’s patch microcirculation	Lymphocyte rolling frequency and rolling velocity; number of sticking (firm-adherent) lymphocytes; transendothelial-migration ratio	Indirect	[103]
	Rolling and endothelial interactions; Firm adhesion and integrin activation	T cells	Autogramin-2	Quantitative adhesion assay on ICAM-1-coated and ECM-coated plates; flow cytometry for LFA-1 conformational states	Percentage of T-cell adhesion to ICAM-1 and ECM substrates	Indirect	[104]
	Rolling and endothelial interactions; Firm adhesion and integrin activation	T cells	MβCD-mediated cholesterol-depletion to disrupt lipid rafts; MβCD-cholesterol repletion for raft reconstitution	Stable Jurkat LFA-1 re-expression cell lines; lipid-raft patching confocal fluorescence microscopy; cell-adhesion assays on ICAM-1/fibronectin substrates	Degree of co-localization of LFA-1/α4β1 with lipid-raft marker cholera toxin B subunit; T-cell adhesive capacity towards ICAM-1 and fibronectin	Indirect	[105]
	Transendothelial migration and interstitial motility	CD3^+^/CD4^+^ T cells	Exogenous LPA; CCL21 chemokine stimulation	Short-term in vivo homing assay with CFSE-labeled T cells; BioFlux flow-chamber transendothelial-migration model under physiological shear stress	Ratio of T cells outside vs. inside HEV	Indirect	[106]
	Transendothelial migration and interstitial motility	CD4^+^/CD8^+^ T cells	autotaxin (ATX) knockout	Boyden–Transwell in vitro T-cell migration assay	In vitro migratory efficiency of Jurkat T cells; altered lymphocyte recruitment into colonic mucosa upon ATX ablation	Indirect	[107]
	Transendothelial migration and interstitial motility	Th2 cells	CCL17/CCL22 ligand stimulation	Flow-cytometric quantification of surface CCR4; Transwell chemotaxis assay	Percentage of cell-surface CCR4 internalization	Indirect	[108]
Mitochondrial Metabolic Reprogramming	Chemokine receptor expression and chemotaxis; Firm adhesion and integrin activation	CD4^+^/CD8^+^ T cells	SDF-1α/CXCL12; pannexin 1; P2X4	Flow-cytometry quantification of graft-infiltrating lymphocytes 24 h post-transplant	T-cell migration speed and migration range	Indirect	[109]
	Chemokine receptor expression and chemotaxis; Firm adhesion and integrin activation	T cells	CXCL12/CCL21; mitochondrial F_1_F_0_-ATP synthase	Transwell chemotaxis towards CXCL12/CCL21/fMLP gradients; migrated cells quantified by flow cytometry.	DRP1 overexpression boosts uropod accumulation and elevates chemotaxis; Promoting mitochondrial fusion strongly inhibits chemotaxis	Direct	[110]
	Rolling and endothelial interactions	CD4^+^, CD8^+^ T cells	S1P	Competitive T-cell adoptive co-transfer	Numbers of naive T cells in secondary lymphoid organs; T-cell thymic-egress and lymph-node-trapping phenotypes	Indirect	[111]
	Transendothelial migration and interstitial motility	CD8^+^ T cells	Co-culture-driven mitochondrial transfer from bone marrow mesenchymal stem cells to CD8^+^ T cells; ethidium bromide treatment of BMSCs to generate functionally deficient donor mitochondria	Transwell co-culture system; flow-sorted Mito^+^/Mito^−^ cells	Quantity of tumor-infiltrating T cells	Indirect	[112]
	Transendothelial migration and interstitial motility	CD8^+^ T cells	Exogenous PGE_2_ stimulation	Mitotracker-Green, tetramethylrhodamine methyl ester mitochondrial dyes	Mitochondrial mass and membrane potential	Indirect	[113]
Crosstalk	Chemokine receptor expression and chemotaxis	CD8^+^ T cells	GPR183; GPR84; GPR3; GPR18	Transwell chemotaxis and competitive co-adoptive-transfer experiments	In vitro chemotaxis efficiency towards tumor-conditioned-media/metabolites	Direct	[114]
	Chemokine receptor expression and chemotaxis	CD4^+^/CD8^+^ T cells; Treg	Traumatic-brain-injury model; acetate–propionate–butyrate SCFAs mixture supplementation	Flow cytometry of brain and intestinal-lamina-propria lymphocytes	Absolute counts of brain-infiltrating CD3^+^, CD4^+^, CD8^+^ and Treg subsets; lamina-propria intestinal T-cell subset enumeration	Indirect	[115]
	Chemokine receptor expression and chemotaxis	T cell	Urocanic acid	Transwell MDSC chemotaxis assay; in vitro HUVEC endothelial-cell culture	MDSC Transwell chemotaxis capacity; endothelial CXCL1 transcript and protein secretion; IκBα-Ser32 phosphorylation status	Direct	[116]
	Chemokine receptor expression and chemotaxis	Th17 cells	Kynurenic acid	Quantity of Th17 cells tissue infiltration	The migration of Th17 cells from mesenteric lymph nodes to the spinal cord	Indirect	[117]
	Firm adhesion and integrin activation	CD8^+^ T cells	GLUT1	Transwell in vitro T-cell migration system	Quantity and spatial distribution of CD8^+^ T cells; frequency of CXCR6^+^ CD8^+^ T cells; in vitro CD8^+^ T-cell migration index	Indirect	[118]

#### 3.2.1. Chemokine Receptor Expression and Chemotaxis

At the level of chemokine receptor expression and chemotaxis, direct experiments demonstrating that amino acid metabolism alters T-cell chemotactic capacity are currently lacking. Sinclair et al. [81] found that autophagy-mediated mitochondrial pruning in naive CD8^+^T cells supports the expression of migration-related molecules, whereas antigen receptor and inflammatory cytokine signaling suppress autophagy by regulating amino acid transporter expression, which may, in turn, indirectly affect migratory capacity; however, this study did not directly perform chemotaxis assays and therefore constitutes indirect evidence. At the level of receptor expression, multiple studies suggest that amino acid metabolism can influence the chemokine receptor repertoire through signaling or epigenetic mechanisms. For example, L-tryptophan induces Treg cells to express the colon-homing receptor GPR15 through AhR signaling, thereby potentially promoting their migratory propensity toward the intestine [82]; death-associated protein kinase 1 (DAPK1) inhibits CD62L and CCR7 expression by activating mTORC1 and promoting the exit of effector T cells from the lymphoid homing program [83]; and citrulline-reactive T cells in early rheumatoid arthritis upregulate CCR6 and CXCR3, which may accelerate their joint infiltration [84]. Furthermore, fumarate can impair the migratory capacity of T helper 17 (Th17) cells toward the central nervous system by inducing DNA hypermethylation of the CCR6 promoter [85], providing indirect evidence that downstream products of amino acid metabolism regulate chemokine receptor expression through epigenetic mechanisms. Collectively, these findings indicate that amino acid metabolism can alter the chemokine receptor expression profile of T cells through multiple pathways; however, most studies have measured only receptor expression or transcriptional changes, which cannot yet be fully equated with alterations in in vivo trafficking function.

#### 3.2.2. Rolling and Endothelial Interactions

Evidence at the rolling and endothelial interaction stage is even weaker, and no functional experiments have directly addressed the relationship between amino acid metabolism and T-cell rolling. Indirect evidence mainly derives from alterations in adhesion molecule expression: a study showed that glutamine supplementation in a murine model of acute colitis downregulates the surface expression of PSGL-1, LFA-1 (CD11a), and the chemokine receptor CCR9 on CD4^+^ Th cells, suggesting that glutamine may indirectly inhibit T-cell rolling or initial adhesion by altering the adhesion molecule profile; however, this was not verified by flow chamber or rolling assays [86]. Furthermore, myeloid-derived suppressor cells (MDSCs) can generate nitric oxide through arginine metabolism, inhibiting endothelial E-selectin expression and thereby potentially attenuating T-cell rolling [87]; however, the effector cells in this context are MDSCs rather than T cells themselves. Glutamine can also promote CCL5 production by epithelial cells through alanine–serine–cysteine transporter 2 (ASCT2)-mediated uptake, thereby influencing T-cell infiltration [88], but this pathway likewise reflects indirect regulation within the microenvironment.

#### 3.2.3. Firm Adhesion and Integrin Activation

Direct experimental evidence for adhesion is also lacking at the firm adhesion and integrin activation stage. Existing indirect evidence includes the following: β1-integrin binding to vascular Cell Adhesion Molecule 1 (VCAM-1) can trigger a glutamine-dependent signaling pathway that stimulates glutamate release from Th17 cells, and this process can be blocked by glutaminase inhibitors or potassium voltage-gated channel subfamily A member 3 (KV1.3) channel inhibitors, suggesting coupling between integrin signaling and glutamine metabolism; however, firm adhesion itself was not directly measured [89]. Another study showed that glutamate receptor activation enhances integrin-mediated adhesion of T cells to extracellular matrix components, providing indirect support for the involvement of amino acid-related metabolites in adhesion regulation [90]. Furthermore, IL-33-induced alterations in arginine metabolism are associated with transcriptional changes in adhesion-related genes in tissue-resident-like Th2 cells, representing evidence at the transcriptional level [91]. Therefore, the regulatory mechanisms by which amino acid metabolism influences firm adhesion remain unclear.

#### 3.2.4. Transendothelial Migration and Interstitial Motility

At the stages of transendothelial migration and interstitial motility, relatively clear direct evidence exists. Simula et al. [92] demonstrated that by using 3D collagen gels and tumor slice models, human CD8^+^ T-cell migration in three-dimensional environments depends primarily on mitochondrial oxidation of glucose and glutamine, rather than fatty acids, and that both mitochondrial ATP and reactive oxygen species (ROS) are required for migration. Pharmacological interventions that enhance mitochondrial activity improve intratumoral migration and the recruitment of CAR T cells to tumor islets. This study directly measured T-cell interstitial motility and represents one of the few pieces of direct evidence that amino acid metabolism regulates trafficking; however, its core contribution lies in the mitochondrial oxidation of glutamine rather than other branches of amino acid metabolism. In terms of indirect evidence, ASCT2-mediated glutamine uptake promotes CCL5 production by epithelial cells through the ROS-signal transducer and activator of the transcription 3 (STAT3) pathway, thereby facilitating T-cell migration [88]; however, this effect does not represent direct regulation of migration by T-cell-intrinsic metabolism. Furthermore, autophagy-mediated mitochondrial pruning supports the expression of migration-related molecules and may also indirectly influence interstitial motility [81].

**Figure 3:**
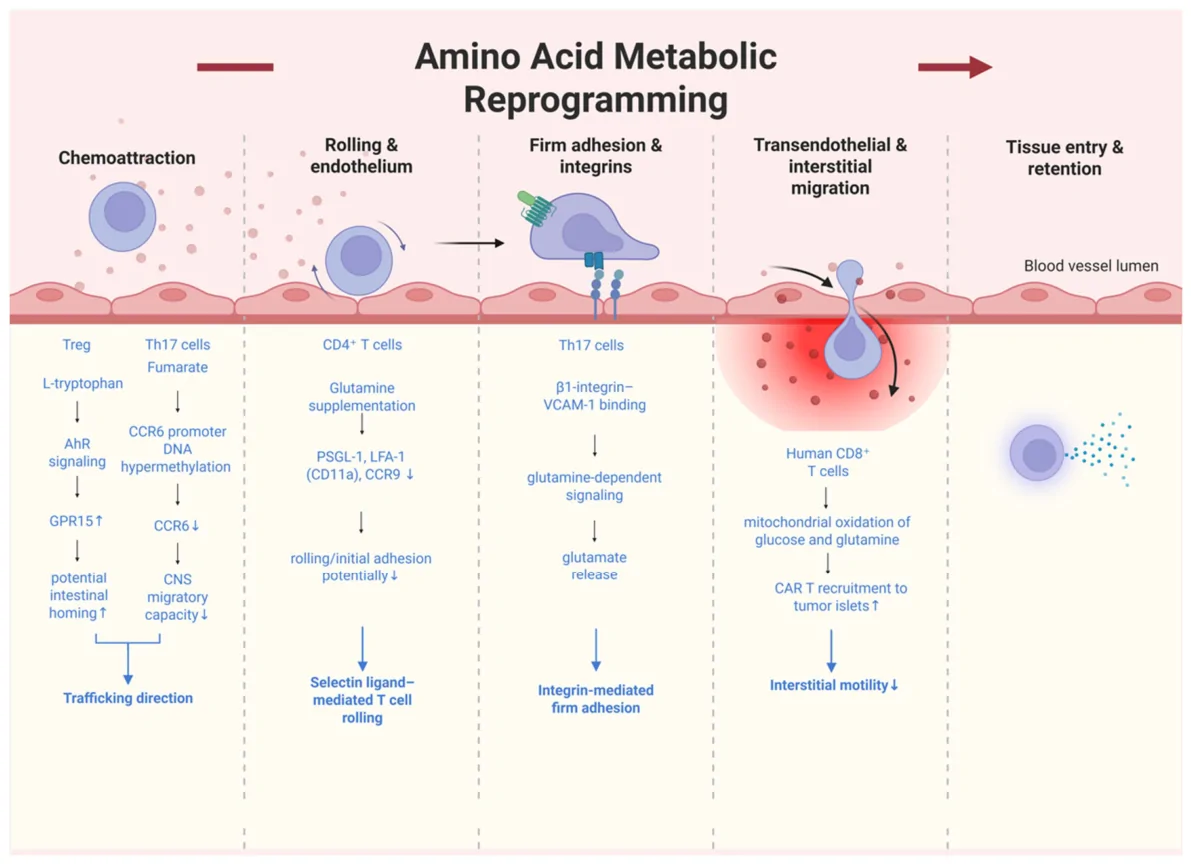
Schematic diagram of amino acid metabolic reprogramming of regulatory nodes in T-cell trafficking. This schematic illustrates the stepwise regulatory mechanisms by which amino acid metabolic reprogramming governs T-cell transendothelial trafficking. The migratory process is divided into four stages, each exemplified by distinct T-cell subsets and their corresponding regulatory pathways. In the chemotaxis stage, regulatory T cells and Th17 cells are exemplified: L-tryptophan upregulates the colon-homing receptor GPR15 through the AhR signaling pathway, potentially enhancing the intestinal homing capacity of regulatory T cells; fumarate mediates DNA hypermethylation of the CCR6 promoter and downregulates CCR6 expression, thereby impairing the migratory capacity of Th17 cells toward the central nervous system. Together, these mechanisms regulate the direction of T-cell trafficking. In the rolling and endothelial interaction stage, CD4^+^T cells are exemplified: glutamine supplementation reduces the surface expression of PSGL-1, LFA-1 (CD11a), and CCR9, potentially attenuating cell rolling and initial adhesion capacity, although this effect has not yet been directly validated by flow chamber or rolling assays. In the firm adhesion and integrin activation stage, Th17 cells are exemplified: β1 integrin binding to VCAM-1 can activate a glutamine-dependent signaling pathway and mediate glutamate release, a process that can be blocked by glutaminase or KV1.3 inhibitors. In the transendothelial migration and interstitial motility stage, human CD8^+^ T cells are exemplified: their three-dimensional migration depends on mitochondrial oxidation of glucose and glutamine rather than fatty acid oxidation, requiring mitochondrial ATP and reactive oxygen species; pharmacological enhancement of this metabolic pathway improves intratumoral migration and increases the recruitment of CAR-T cells to tumor islets. Arrows indicate the direction of metabolic pathways/regulatory cascades and denote promotion, activation, or downstream effects; the symbol ↓ indicates downregulation or decreased activity.

Overall, the regulation of T-cell trafficking by amino acid metabolism is supported by relatively clear direct evidence in interstitial motility, whereas the chemotaxis, rolling and firm adhesion stages remain dominated by indirect evidence, with some findings also involving microenvironmental cells or effector functions. Future studies will require more functional experiments that directly measure each trafficking stage to fill these gaps. Future studies will require more functional experiments that directly measure each trafficking stage to fill these gaps. The stage-specific roles and regulatory mechanisms of amino acid metabolic reprogramming in T-cell trafficking are summarized in Figure 3.

### 3.3. Lipid Metabolic Reprogramming

Lipid metabolic reprogramming refers to the systematic reorganization of lipid uptake, de novo synthesis, storage, lipolysis, fatty acid oxidation, cholesterol/sphingolipid metabolism, and lipid signaling networks in T cells upon activation, differentiation, and microenvironmental stimulation, in order to meet the demands for energy supply, membrane structural remodeling, and signal transduction. In T-cell trafficking, such reprogramming not only provides the energy required for migration but also dynamically regulates T-cell chemotaxis, rolling, firm adhesion and transendothelial migration by remodeling plasma membrane lipid composition and lipid raft microdomains, modulating chemokine receptor and integrin function, and influencing cytoskeletal rearrangement and endothelial adhesion.

#### 3.3.1. Chemokine Receptor Expression and Chemotaxis

At this stage, several direct functional lines of evidence are available. Cucchi et al. [93] demonstrated in in vitro chemotaxis assays that treatment of activated CD4^+^T cells with eicosapentaenoic acid (EPA) and docosahexaenoic acid (DHA) significantly reduces their chemotactic motility, and different n-3 polyunsaturated fatty acids (PUFAs) exhibit distinct chemotactic properties. Van Gijsel-Bonnello et al. [94] found that pantethine treatment of multiple T-cell types downregulates CXCL12-driven chemotaxis, with mechanisms involving altered membrane dynamics of CXCR4 and C-X-C Chemokine Receptor Type 7 (CXCR7) and changes in cellular redox state. Amersfoort et al. [95] demonstrated that a synthetic peroxisome proliferator-activated receptor delta (PPARδ) agonist increases the in vitro migratory capacity of Treg cells in a fatty acid oxidation-dependent manner. These studies directly measured the chemotactic or migratory behavior of T cells.

At the level of receptor expression and signal integration, the PI3K–Foxo axis controls CCR7 and CD62L expression through regulation of Kruppel-like factor 2 (KLF2), thereby influencing naive T-cell migration to secondary lymphoid organs; this signaling axis is regulated by the lipid second messenger phosphatidylinositol 3,4,5-trisphosphate (PI(3,4,5)P3) and can be regarded as a signaling hub between lipid metabolic status and trafficking receptor expression9 [96,97,98]. Retinoic acid, a metabolite of vitamin A, induces T-cell expression of the gut-homing receptor CCR9 and α4β7 integrin through RARα signaling, serving as a typical example of lipid-soluble metabolites regulating chemokine receptor expression [99,100]. Under obese or high-fat diet conditions, hepatic steatosis upregulates CXCL12 and CXCL13 while enhancing the sensitivity of T cells and B cells to chemotactic signals, collectively driving pathological lymphocyte migration to the liver [101]; however, these observations primarily reflect the indirect influence of systemic lipid metabolic dysregulation on the chemotactic microenvironment.

#### 3.3.2. Rolling and Endothelial Interactions

Murai et al. [102] directly measured T-cell rolling adhesion under physiological flow conditions and found that depletion of membrane cholesterol with methyl-β-cyclodextrin (MβCD) upregulated the hyaluronan-binding ability of CD44 and increased the number of rolling adherent cells. This indicates that disruption of lipid raft structure can alter the functional state of Cluster of Differentiation 44 (CD44); however, it does not suggest that lipid metabolism as a whole promotes or inhibits rolling, but rather that the integrity of membrane cholesterol and lipid rafts participates in rolling regulation. Earlier, in 1997, Tsuzuki et al. directly observed through intravital fluorescence microscopy that in the postcapillary venules of rat Peyer’s patches, olive oil gavage significantly enhanced lymphocyte rolling and adhesion, and markedly increased T lymphocyte transendothelial migration. Olive oil enhanced the expression of α4 integrin and L-selectin on lymphocytes, whereas caprylic acid only increased L-selectin expression and stimulated lymphocyte rolling without affecting adhesion [103]. This suggests that dietary fat components can differentially regulate rolling by altering the expression of adhesion molecules on the lymphocyte surface, providing indirect evidence for the association between T-cell rolling and lipid metabolism.

#### 3.3.3. Firm Adhesion and Integrin Activation

A study reported that treatment of effector T cells with the synthetic sterol autogramin-2 inhibits LFA-1-dependent adhesion to ICAM-1 and extracellular matrix within 30 min [104]. Mechanistically, autogramin-2 rapidly stimulates lipolysis, and the released fatty acids form acylcarnitines with carnitine, inducing plasma membrane remodeling and causing LFA-1 to be excluded from lipid rafts. Van Gijsel-Bonnello et al. [94] also demonstrated that pantethine treatment downregulates T-cell adhesion, providing direct experimental evidence for adhesion. Lipid raft microdomains enriched in cholesterol and sphingolipids can promote the oligomerization of LFA-1 and α4β1 integrins, enhance their binding to endothelial ligands ICAM-1 and VCAM-1, and drive the transition from low-affinity rolling to high-stability adhesion [105], a mechanism that provides indirect support for the regulation of firm adhesion by lipid metabolism.

#### 3.3.4. Transendothelial Migration and Interstitial Motility

At this stage, several direct lines of experimental evidence are available. Cucchi et al. [93] demonstrated in transendothelial migration assays that CD4^+^T cells treated with EPA and DHA exhibited significantly reduced chemotactic motility, and that after adoptive transfer into recipient mice, the migration efficiency of treated T cells toward the inflamed peritoneum was also significantly decreased. Van Gijsel-Bonnello et al. [94] demonstrated that pantethine treatment downregulates transendothelial migration of multiple T-cell types. Amersfoort et al. [95] reported that a PPARδ agonist increases Treg migratory capacity in a fatty acid oxidation-dependent manner. These studies directly measured T-cell transendothelial migration or in vivo migration. Furthermore, lysophosphatidic acid (LPA), catalyzed by B-cell-derived autotaxin, can promote cytoskeletal rearrangement and endothelial barrier penetration by activating the extracellular signal-regulated kinase (ERK) signaling pathway in T cells [106,107]; however, the relationship between LPA production and metabolic reprogramming remains to be further clarified. The chemokine CCL22 can induce lipid raft-dependent internalization of CCR4, spatiotemporally terminating migratory signals to ensure completion of the extravasation process [108], which constitutes indirect evidence for the regulation of transendothelial migration by lipid rafts.

**Figure 4:**
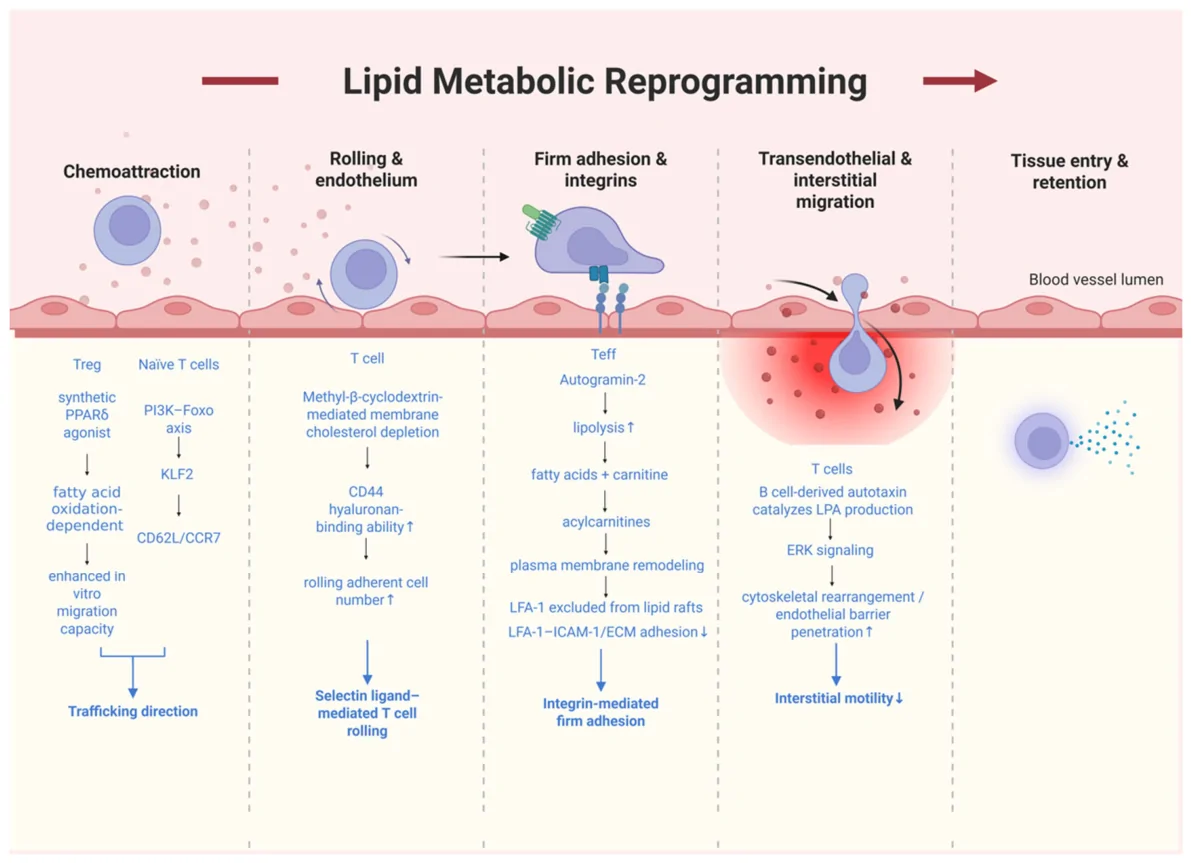
Schematic diagram of lipid metabolic reprogramming of regulatory nodes in T-cell trafficking. This schematic illustrates the stepwise regulatory mechanisms by which lipid metabolic reprogramming governs the entire process of T-cell transendothelial trafficking. The migratory process is divided into four stages, each exemplified by distinct T-cell subsets and their corresponding regulatory pathways. In the chemotaxis stage, regulatory T cells and naive T cells are exemplified: a synthetic PPARδ agonist enhances the in vitro migratory capacity of regulatory T cells through a fatty acid oxidation-dependent pathway; in naive T cells, the PI3K–Foxo axis regulates the expression of CCR7 and CD62L through KLF2, mediating their migration to secondary lymphoid organs, and this process is regulated by the lipid second messenger PI(3,4,5)P_3_. Together, these mechanisms determine the direction of T-cell trafficking. In the rolling and endothelial interaction stage, T cells are exemplified: depletion of membrane cholesterol by methyl-β-cyclodextrin enhances the binding capacity of CD44 to hyaluronan and increases the number of rolling adherent cells, a regulatory process involving the integrity of lipid raft structures. In the firm adhesion and integrin activation stage, effector T cells are exemplified: autogramin-2 promotes lipolysis, and the released fatty acids combine with carnitine to generate acylcarnitines, inducing plasma membrane remodeling and causing LFA-1 to be excluded from lipid raft structures, ultimately attenuating the adhesion of LFA-1 to ICAM-1 and the extracellular matrix. In the transendothelial migration and interstitial motility stage, T cells are exemplified: B-cell-derived autotaxin catalyzes the production of lysophosphatidic acid, which activates the ERK signaling pathway in T cells, promoting cytoskeletal rearrangement and enhancing endothelial barrier penetration, ultimately increasing interstitial motility. Arrows indicate the direction of metabolic pathways/regulatory cascades and denote promotion, activation, or downstream effects; the symbol ↓ indicates downregulation or decreased activity.

Overall, the regulation of T-cell trafficking by lipid metabolic reprogramming is supported by several lines of direct functional evidence in the chemotaxis, firm adhesion and transendothelial migration, primarily involving alterations in membrane lipid composition, lipolysis, fatty acid oxidation, and lipid signaling. Direct evidence remains relatively scarce for the rolling steps, and some of the available evidence derives from in vitro experiments or involves microenvironmental cells. Future studies should incorporate flow chamber rolling assays, in vivo competitive homing experiments, and residence kinetics analyses to clarify the specific roles of lipid metabolism at each trafficking step. Future studies should incorporate flow chamber rolling assays, in vivo competitive homing experiments, and residence kinetics analyses to clarify the specific roles of lipid metabolism at each trafficking step. The stage-specific roles and regulatory mechanisms of lipid metabolic reprogramming in T-cell trafficking are summarized in Figure 4.

### 3.4. Mitochondrial Metabolic Reprogramming

Mitochondrial metabolic reprogramming refers to a fundamental cellular mechanism that dynamically adapts mitochondrial function in response to microenvironmental changes. This process encompasses core pathways such as aerobic glycolysis, dynamic regulation of OXPHOS, fatty acid oxidation, glutaminolysis, reactive oxygen species (ROS) signaling, and mitophagy. These metabolic pathways are integrated through epigenetic modifications, transcriptional regulation, and post-translational modifications, collectively establishing a central metabolic hub that enables T cells to efficiently sense and respond to microenvironmental signals.

#### 3.4.1. Chemokine Receptor Expression and Chemotaxis

At this stage, several direct functional lines of evidence are available. Simula et al. [92] treated human CD8^+^ T cells with tricarboxylic acid (TCA) cycle inhibitors and the mitochondrial ATP synthase inhibitor oligomycin and observed in in vitro chemotaxis assays that chemotactic migration was significantly inhibited, with this process being dependent on mitochondrial oxidation of glucose and glutamine, rather than fatty acids. Ledderose et al. [109] demonstrated that stromal-cell-derived factor 1 alpha (SDF-1α)/CXCL12 triggers mitochondrial ATP production, and that inhibition of mitochondrial ATP generation blocks the chemotactic migration of primary human CD4^+^ T cells toward SDF-1α; although this study also involved P2X purinoceptor 4 (P2X4) receptor signaling, the metabolic contribution of mitochondrial ATP is clearly established. Campello et al. [110] reported that mitochondrial respiration is a limiting factor for chemotaxis, and that respiratory chain inhibitors significantly suppress T-cell chemotaxis. Collectively, these results indicate that mitochondrial oxidative metabolism provides fundamental support for T-cell chemotaxis. In terms of indirect evidence, effector CD8^+^ T cells undergo a metabolic shift from mitochondrial OXPHOS to glycolysis upon activation, and this transition is associated with changes in chemotactic capacity; however, this represents a correlative observation between metabolic phenotype and function. Simula et al. [92] further found that the chemotactic motility of CD8^+^ T cells strongly correlates with mitochondrial mass and other parameters of OXPHOS.

#### 3.4.2. Rolling and Endothelial Interactions

Currently, direct functional experiments examining the effect of mitochondrial metabolic pathways on the T-cell rolling step are lacking. Indirect evidence derives mainly from the microenvironmental level: lymphatic endothelial-cell-derived sphingosine-1-phosphate (S1P) signaling can maintain mitochondrial content and function in naive T cells, providing energy for migration; however, this effect is not T-cell-intrinsic metabolic reprogramming, but rather a non-cell-autonomous process in which endothelial-derived signals act on T cells [111]. Therefore, the regulation of the T-cell rolling step by mitochondrial metabolism still requires dedicated flow chamber experiments to verify.

#### 3.4.3. Firm Adhesion and Integrin Activation

At this stage, direct experimental evidence is available. Simula et al. [92] found that treatment with TCA cycle inhibitors or oligomycin significantly inhibited the spreading and adhesion capacity of CD8^+^ T cells, and CRISPR/Cas9-mediated double gene editing of pyruvate dehydrogenase E1 alpha subunit (PDHA1) and oxoglutarate dehydrogenase (OGDH) also impaired T-cell adhesion, directly demonstrating that mitochondrial oxidative metabolism supports integrin-mediated firm adhesion. In terms of indirect evidence, lung resident memory T-cell subsets defined by integrin expression are metabolically distinguishable, and skin-derived memory T cells exhibit higher levels of fatty acid oxidation and OXPHOS than circulating memory cells, suggesting that mitochondrial metabolism may be associated with adhesion-related phenotypes.

#### 3.4.4. Transendothelial Migration and Interstitial Motility

This stage is supported by the most abundant and direct functional evidence. Simula et al. [92] demonstrated using three-dimensional collagen gels and tumor slice models that the TCA cycle is the principal metabolic pathway maintaining the three-dimensional motility of human CD8^+^ T cells, with glycolysis playing only a secondary role. CD8^+^ T-cell migration depends on mitochondrial oxidation of glucose and glutamine, but not fatty acids, and both mitochondrial ATP and ROS are required for migration. The study further showed that pharmacological interventions that enhance mitochondrial activity, including rapamycin, 5-aminoimidazole-4-carboxamide ribonucleoside (AICAR), and β-nicotinamide adenine dinucleotide (β-NAD), significantly increase the three-dimensional motility of CD8^+^ T cells. In addition, the three-dimensional motility of anti-epidermal growth factor receptor (EGFR) CD8^+^ CAR T-cells similarly depends on mitochondrial metabolism supported by glucose and glutamine, and CRISPR/Cas9-mediated double gene editing of PDHA1 and OGDH significantly reduces their three-dimensional motility. In transendothelial migration assays, rapamycin-pretreated CAR T cells exhibited enhanced transendothelial migratory capacity; in vivo experiments demonstrated that enhancing mitochondrial metabolism improves intratumoral migration of CD8^+^ T cells and the recruitment of CAR T cells to tumor islets. These findings directly measured interstitial motility, transendothelial migration, and in vivo migration. In terms of indirect evidence, intercellular nanotube-mediated mitochondrial transfer can enhance T-cell metabolic fitness and antitumor efficacy, but this represents a non-T-cell-autonomous effect [112]; prostaglandin E2 (PGE_2_) in the intestinal microenvironment can induce mitochondrial depolarization in CD8^+^ T cells, which also constitutes a microenvironmental factor rather than T-cell-intrinsic metabolic reprogramming [113].

**Figure 5:**
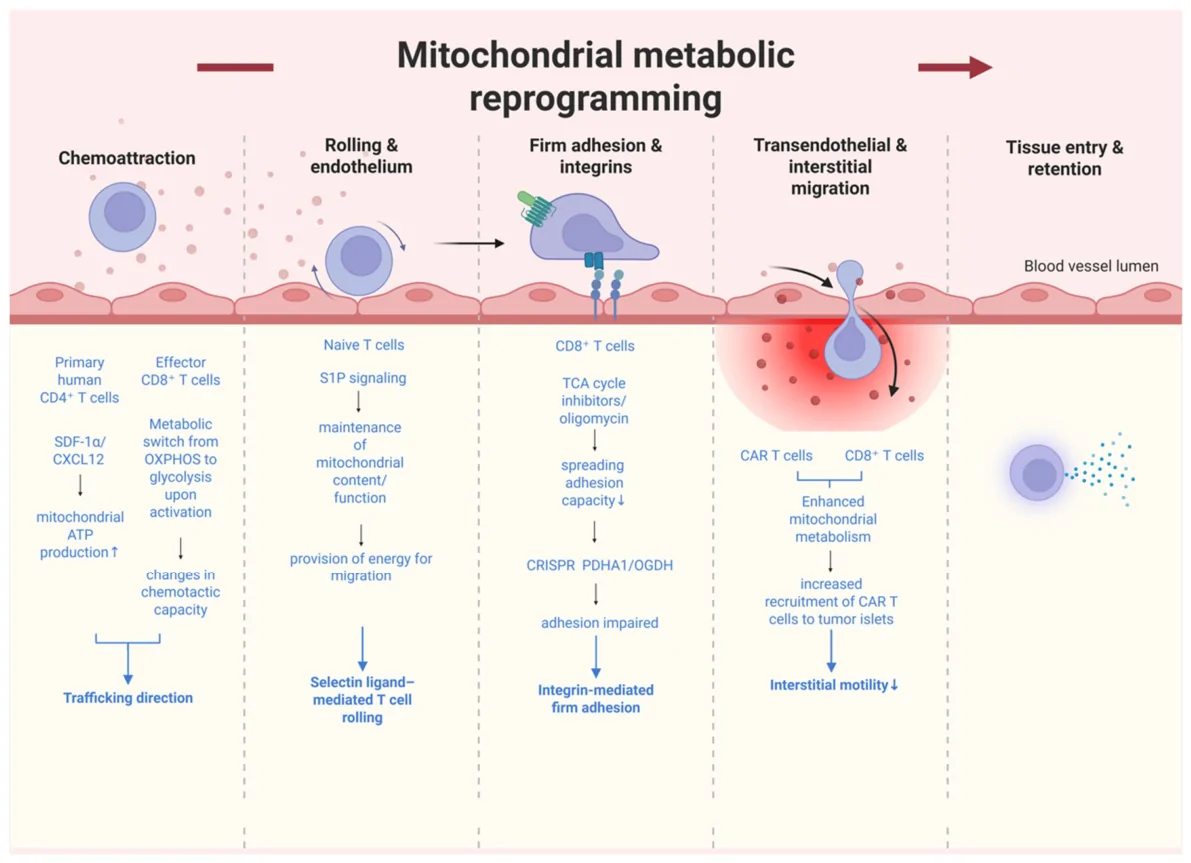
Schematic diagram of mitochondrial metabolic reprogramming of regulatory nodes in T-cell trafficking. This schematic illustrates the stepwise regulatory mechanisms by which mitochondrial metabolic reprogramming governs the entire process of T-cell transendothelial trafficking. The migratory process is divided into four stages, each exemplified by distinct T-cell subsets and their corresponding regulatory pathways. In the chemotaxis stage, primary human CD4^+^ T cells and effector CD8^+^ T cells are exemplified: SDF-1α/CXCL12 can promote mitochondrial ATP production in primary CD4^+^ T cells; effector CD8^+^ T cells undergo a metabolic switch from OXPHOS to glycolysis upon activation, thereby altering their chemotactic capacity. Together, these mechanisms regulate the direction of T-cell trafficking. In the rolling and endothelial interaction stage, naive T cells are exemplified: lymphatic endothelial-cell-derived S1P signaling can maintain mitochondrial content and function, providing energy for cell migration and supporting the selectin ligand-mediated rolling process. In the firm adhesion and integrin activation stage, CD8^+^ T cells are exemplified: treatment with TCA cycle inhibitors or oligomycin reduces cell spreading and adhesion capacity; CRISPR editing of PDHA1/OGDH genes similarly impairs cell adhesion function, confirming that mitochondrial metabolism participates in regulating integrin-mediated firm adhesion. In the transendothelial migration and interstitial motility stage, CD8^+^ T cells and CAR-T cells are exemplified: enhanced mitochondrial metabolism can increase the recruitment efficiency of CAR-T cells to tumor islets, promoting interstitial motility and tissue infiltration. Arrows indicate the direction of metabolic pathways/regulatory cascades and denote promotion, activation, or downstream effects; the symbol ↓ indicates downregulation or decreased activity.

Overall, the regulation of T-cell trafficking by mitochondrial metabolic reprogramming is supported by relatively clear direct functional evidence in chemotaxis, firm adhesion, transendothelial migration, and interstitial motility, with the systematic study by Simula et al. [94] being the most prominent. The rolling steps remain dominated by indirect evidence or microenvironmental influences. Future studies should further distinguish the roles of mitochondrial metabolic pathways per se from those of mitochondrial dynamics and mitochondrial signaling, and should supplement direct trafficking experiments for the rolling and retention steps. Future studies should further distinguish the roles of mitochondrial metabolic pathways per se from those of mitochondrial dynamics and mitochondrial signaling, and should supplement direct trafficking experiments for the rolling and retention steps. The stage-specific roles and regulatory mechanisms of mitochondrial metabolic reprogramming in T-cell trafficking are summarized in Figure 5.

### 3.5. Crosstalk

Metabolic crosstalk represents a fundamental biological process within multicellular systems, wherein different cell types dynamically regulate functional outputs and microenvironmental homeostasis through metabolite exchange, pathway coordination, and resource competition. Its core mechanisms encompass five synergistic modes: (1) reciprocal metabolite exchange forming the material basis; (2) metabolic complementarity enabling cross-cellular pathway cooperation; (3) metabolic competition modulating resource allocation; (4) metabolic reprogramming allowing dynamic adaptation to environmental stress; and (5) metabolite signaling amplifying regulatory effects. Collectively, these mechanisms underpin physiological processes such as tissue development and immune regulation by driving metabolic network remodeling through reprogramming, maintaining a complementarity–competition equilibrium, and extending signal-mediated control.

#### 3.5.1. Chemokine Receptor Expression and Chemotaxis

At the chemotaxis stage, Kim et al. [114] identified metabolite-sensing receptors including GPR183, GPR84, GPR34, and GPR18 through a CRISPR activation screen, and demonstrated that T cells expressing these receptors undergo chemotactic migration in response to tumor-cell-derived metabolites such as 7α,25-dihydroxycholesterol. This study includes both in vitro and in vivo chemotaxis assays and currently represents the most direct evidence for intercellular metabolite signaling. Another study showed that supplementation with short-chain fatty acids (SCFAs) after traumatic brain injury increases the infiltration of Treg and Th1 cells into brain tissue, and that gut T cells are one of the cellular sources of brain-infiltrating cells, suggesting that gut microbiota-derived SCFAs influence the migratory propensity of T cells toward the brain via systemic circulation [115]. However, this study primarily reports changes in tissue infiltration numbers without direct chemotaxis measurements, and therefore can be considered a relatively weak form of direct evidence. Furthermore, the microbial metabolite urocanic acid, produced by Muribaculum, can act on inhibitor of nuclear factor kappa B alpha (IκBα) in tumor vascular endothelial cells to reduce CXCL1 secretion, thereby decreasing MDSC recruitment and increasing CD8^+^T-cell activation; this process represents a three-level crosstalk from metabolites to endothelial cells and then to immune cells, and the step ultimately affecting T-cell infiltration involves changes in chemokines [116]. In terms of indirect evidence, kynurenic acid, a microbial metabolite produced by Sporosarcina pasteurii, can recruit macrophages to mesenteric lymph nodes through the GPR35 receptor, and these macrophages subsequently secrete IL-6 to promote the migration of Th17 cells from mesenteric lymph nodes to the spinal cord [117]. This evidence represents a cascade crosstalk from metabolites to macrophages and then to T cells, but does not directly measure Th17 chemotactic function.

#### 3.5.2. Rolling and Endothelial Interactions

Currently, functional evidence that metabolic crosstalk directly regulates T-cell rolling in accordance with a strict definition is lacking. Although it has been reported that SCFAs can increase L-selectin surface expression and mRNA levels on neutrophils to stimulate their migration (short-chain fatty acids stimulate the migration of neutrophils to inflammatory sites); this phenomenon occurs in neutrophils rather than T cells and cannot be extrapolated to the T-cell rolling step. Therefore, this step can currently only be regarded as an evidence gap.

#### 3.5.3. Firm Adhesion and Integrin Activation

Similarly, experimental reports directly examining the effect of metabolic crosstalk on integrin-mediated firm adhesion of T cells are lacking. In existing studies, glycolytic cancer-associated fibroblasts secrete CXCL16 in a GLUT1-dependent manner to trap CD8^+^T cells at the tumor margin [118]; however, the direct signal between cancer-associated fibroblasts and T cells is the chemokine CXCL16 rather than the metabolite itself, and this can therefore only serve as indirect contextual evidence that metabolic status influences the chemokine environment, rather than as evidence of crosstalk at the firm adhesion step.

#### 3.5.4. Transendothelial Migration and Interstitial Motility

Evidence that metabolic crosstalk directly regulates T-cell transendothelial migration or interstitial motility is currently still lacking. Existing studies mostly involve intercellular communication mediated by chemokines or cytokines, rather than metabolites themselves acting as signals [119]. Therefore, no well-supported conclusion can be drawn at this stage.

Overall, direct evidence that metabolic crosstalk regulates T-cell trafficking is currently concentrated mainly in the chemotaxis stage (with GPR183 sensing of tumor metabolites being the most clearly defined). The rolling, firm adhesion, and transendothelial migration stages still lack direct evidence that meets the strict definition. Future studies should focus on the role of metabolites as intercellular signals in the early stages of trafficking and should employ direct functional approaches such as chemotaxis assays, flow chambers, transendothelial migration assays, and intravital imaging for validation.

## 4. Discussion

This review systematically examines the regulatory roles of glucose metabolism, amino acid metabolism, lipid metabolism, and mitochondrial metabolic reprogramming at each stage of T-cell trafficking (chemotaxis, rolling, firm adhesion, transendothelial migration and interstitial motility), and provides a graded assessment of the existing literature according to direct versus indirect evidence. Integrated analysis reveals that the depth of regulation and strength of evidence vary markedly across metabolic types and trafficking stages, and that the field still faces several important challenges in terms of conceptual boundaries, research coverage, and translational application.

Taken together, the distribution of evidence across the four metabolic types leads to the following core conclusions. Mitochondrial metabolic reprogramming has the most robust evidence at the interstitial motility stage, where the systematic study by Simula et al. [92] employed multiple approaches including three-dimensional collagen gels, tumor slice models, gene editing, and pharmacological interventions to demonstrate that the TCA cycle is the principal metabolic pathway maintaining the three-dimensional motility of human CD8^+^ T cells, and that this process depends on mitochondrial oxidation of glucose and glutamine rather than fatty acids. Glucose metabolic reprogramming has relatively concentrated direct evidence at the chemotaxis and firm adhesion stages, mainly involving glycolysis supporting chemotactic migration and integrin-dependent adhesion. Evidence for lipid metabolic reprogramming in regulating chemotaxis and transendothelial migration is more dispersed, primarily derived from changes in membrane lipid composition and PPARδ-mediated fatty acid oxidation. Direct evidence for amino acid metabolic reprogramming is the most limited, focusing mainly on the support of interstitial motility by glutamine oxidation.

In terms of trafficking stages, interstitial motility is the stage with the most abundant evidence, with all four metabolic types supported to varying degrees by direct evidence. Chemotaxis and transendothelial migration come next. Evidence at the rolling and firm adhesion stages is the weakest, with direct functional experiments for each metabolic type at these two stages being very limited. Furthermore, this review also covers the special dimension of metabolic crosstalk, namely the indirect regulation of T-cell trafficking through metabolite exchange and metabolite signaling between different cell types. However, direct evidence for intercellular metabolic crosstalk that meets a strict definition remains scarce, at the chemotaxis stage, there is also evidence that metabolite-sensing receptors such as GPR183 enable T cells to directly sense tumor metabolites, but the rolling, firm adhesion, and transendothelial migration stages are essentially devoid of evidence. This distribution pattern may reflect differences in the degree to which different trafficking stages depend on metabolism, or it may reflect differences in the experimental accessibility of each stage with current research tools. Wider adoption of techniques such as flow chamber assays and intravital imaging is expected to fill these gaps.

The regulation of immune cell migration by metabolism is not unique to T cells but represents a general biological phenomenon. Marelli-Berg and Jangani have pointed out that leukocyte metabolism may undergo reprogramming in response to migratory stimuli and the different environmental signals received during circulation, ultimately regulating leukocyte motility and migration [120]. The review by Guak and Krawczyk also systematically elaborates on the relationship between cellular metabolism and immune cell migration [75]. Metabolic regulation of dendritic cell homing is well supported by research: Liu et al. reported that CCR7 stimulation activates the HIF-1α pathway in dendritic cells, leading to metabolic reprogramming toward glycolysis and thereby driving their migration [121]; Guak et al. further confirmed that inhibition of glycolysis impairs CCR7 oligomerization and migration of dendritic cells to draining lymph nodes [122]. The metabolism of B cells differs significantly from that of T cells, as Nakano et al. found that T cells and B cells adopt distinct fatty acid utilization strategies [123]. NK cells and macrophages are likewise regulated by metabolism; the review by Ghosh et al. systematically describes the regulatory role of mitochondrial dynamics and metabolic attributes in NK cell function and infiltration in the tumor microenvironment [124]. Nevertheless, T cells remain the most deeply and systematically studied model in this field.

The discussion in this review is primarily based on knowledge of adaptive αβ T cells. Whether the trafficking of non-antigen-specific T-cell subsets such as invariant T cells (e.g., invariant natural killer T cells or mucosal-associated invariant T cells) and γδ T cells is differentially regulated by metabolic reprogramming remains largely unstudied. These subsets differ substantially from αβ T cells in tissue distribution, metabolic features, and migration patterns, and their mechanisms of metabolic regulation of trafficking may be unique. This knowledge gap limits a comprehensive understanding of the metabolic regulation of T-cell trafficking. Evidence regarding whether costimulatory and coinhibitory receptors expressed on the surface of T cells regulate trafficking through metabolic reprogramming is still limited. The study by Kishore indicated that CD28 promotes Treg migration by inducing glycolysis, whereas cytotoxic T-lymphocyte-associated protein 4 inhibits Treg migration by suppressing glycolytic enzymes [74]. CD31 can selectively modulate the migratory response of activated T cells to inflammatory chemokines. However, much of this evidence comes from indirect reasoning, and direct functional experiments are still lacking.

At the translational application level, drugs targeting T-cell trafficking itself have achieved significant clinical translation. In the field of integrin targeting, natalizumab received U.S. Food and Drug Administration (FDA) approval in 2004 for the treatment of relapsing-remitting multiple sclerosis, acting by blocking α4 integrin-mediated trafficking of pathogenic effector T cells in brain tissue. Vedolizumab received FDA approval in 2014 for the treatment of moderate-to-severe ulcerative colitis and Crohn’s disease, selectively blocking the interaction between α4β7 integrin and intestinal endothelial MAdCAM-1 to specifically inhibit the trafficking of intestinal lymphocytes. In 2024, the FDA further approved the subcutaneous formulation of vedolizumab for maintenance therapy of Crohn’s disease. In the field of S1P receptor modulators, fingolimod received FDA approval in 2010, inducing internalization and degradation of S1P1 receptors to retain lymphocytes in secondary lymphoid organs, thereby inhibiting the migration of autoreactive T cells to the central nervous system. Newer-generation S1P receptor modulators such as ozanimod and siponimod have also been approved for multiple sclerosis. The clinically approved drugs targeting T-cell trafficking are summarized in Table 2.

Compared with the above drugs that directly target trafficking receptors, drug development aimed at regulating T-cell trafficking through metabolic reprogramming is at a critical stage of translation from preclinical to clinical settings. The metabolic inhibitors targeting metabolic reprogramming to regulate T-cell trafficking are summarized in Table 3. Multiple classes of metabolic modulators have shown potential in preclinical and early clinical studies to regulate T-cell migration. In the regulation of glucose metabolism, the glycolytic inhibitor (2-DG) has been evaluated for its effects on T-cell migration in multiple preclinical studies. It was found that 2-DG selectively inhibits glycolysis and controls the migration of terminally differentiated effector memory T cells, re-expressing CD45RA (TEMRA) and EM CD8^+^ T cells, with mechanisms involving impairment of sialic acid biosynthesis and blockade of PSGL-1 conformational transition to a functional structure. However, while inhibiting tumor glycolysis, 2-DG also severely interferes with T-cell proliferation and activation, which constitutes a core obstacle to its clinical translation. Metformin, an approved drug for type 2 diabetes, has been shown to directly act on CD8^+^ T cells through adenosine monophosphate-activated protein kinase (AMPK) activation. Adoptive transfer experiments showed that antigen-specific CD8^+^ T cells treated with 10 μM metformin could migrate efficiently to tumors while maintaining multifunctionality, an effect that could be blocked by the AMPK inhibitor compound C [125]. Further studies revealed that metformin can induce CD8^+^ T cells to convert to a memory-like CD8^+^CXCR3^+^ phenotype, the formation of which depends on CXCR3 expression for homing to the lung mucosa. Recent research has also found that metformin combined with periodic fasting–refeeding can enhance tumor infiltration of CXCR6^+^ CD8^+^ T cells through VCAM-1 upregulation.

In the regulation of amino acid metabolism, the glutaminase inhibitor CB-839 (telaglenastat) has been evaluated in multiple preclinical tumor immunology models and has also advanced to randomized clinical trials. Studies have shown that CB-839 has minimal effects on T-cell proliferation, suggesting differences in glutamine utilization pathways between tumor cells and T cells. CB-839 can increase the availability of glutamine to T cells in the tumor microenvironment, thereby enhancing effector T-cell infiltration into tumors. In vivo experiments demonstrated that CB-839 treatment can activate melanoma antigen-specific T cells and improve their tumor-killing activity in adoptive T-cell therapy models in immunocompetent mice. However, results from randomized testing have not supported this expectation. In the phase II CANTATA trial, 444 patients with metastatic clear cell renal cell carcinoma were randomly assigned to receive telaglenastat plus cabozantinib or placebo plus cabozantinib. The trial did not meet its primary endpoint: median progression-free survival was 9.2 months with telaglenastat versus 9.3 months with placebo (hazard ratio 0.94, 95% CI 0.74–1.21, *p* = 0.65) [126]. This negative result indicates that, without patient selection, inhibition of glutamine metabolism may be insufficient to improve T-cell trafficking or tumor control. In addition, the toxicity profile of telaglenastat itself should be distinguished from the severe systemic toxicity reported for broad glutamine antagonist metabolites. In the CANTATA trial, the frequency and severity of adverse events were comparable between the two arms, and other phase I/II studies have reported that telaglenastat-containing regimens are generally well tolerated, with most adverse events being grade 1or 2 [127]. Therefore, the central obstacle for this class of drugs is likely not systemic toxicity but the lack of predictive biomarkers with which to identify tumors that are dependent on glutamine metabolism.

In the regulation of mitochondrial metabolism, the mTOR inhibitor sirolimus (rapamycin) has received clinical approval in transplantation anti-rejection and autoimmune diseases, and has shown potential in regulating T-cell migration. Studies have found that sirolimus significantly inhibits CXCL12-mediated migration of primary human resting T cells and the human T-cell leukemia cell line CEM [128]. Inhibition of mTOR can lead to re-expression of CD62L and CCR7 on effector T cells and their homing to secondary lymphoid organs. Simula et al. [92] further demonstrated that sirolimus pretreatment can increase the maximal respiratory capacity of CD8^+^ CAR T cells without altering basal respiration levels, thereby enhancing their three-dimensional motility and recruitment to tumor islets. However, sirolimus reduces the in vitro expansion rate of CD8^+^ CAR T cells due to inhibition of mTOR-dependent glycolysis. Studies suggest that alternative strategies such as bezafibrate, or the use of nutrients such as lactate, acetate, and pyruvate that bypass glycolysis to directly fuel OXPHOS, may be better options without compromising proliferation. These findings highlight the importance of finding a precise balance between regulating T-cell metabolism to promote migration and maintaining their proliferative capacity.

Emerging technologies are providing revolutionary tools for research on the metabolic regulation of T-cell trafficking. In the field of gene editing, CRISPR-Cas9 technology has become an important means to enhance the tumor infiltration capacity of CAR-T cells [129]. CRISPR activation screening has been used to identify key genes that can enhance immune cell infiltration into solid tumors. Kim et al. [114]. reported in *Nature Immunology* that through in vitro and in vivo CRISPR activation screening, metabolite-sensing G Protein-Coupled Receptors such as GPR183, GPR84, GPR34, and GPR18 were identified as enhancers of NK and T-cell infiltration and chemotaxis toward breast and ovarian tumors. Expression of GPR183 in NK cells, CAR NK cells, and CAR T cells increases tumor infiltration and tumor control. This study demonstrates that metabolite sensing can be reprogrammed to obtain biochemically guided spatially targeted cells, creating new possibilities for therapeutic intervention. This strategy represents a paradigm shift from “passively utilizing chemokine receptors” to “actively designing metabolite sensing.”.

In the engineering modification of CAR-T cells, gene editing technology has been systematically applied in four aspects: CAR-T cells modified with infiltration and migration markers, differentiation into effector T cells to enhance cytokine and cytotoxic functions, blockade of inhibitory receptor ligands and elimination of exhaustion-related transcription, and improvement of metabolic adaptability of CAR-T cells. A study published in *Nature* in 2026 used in vivo genome-wide CRISPR screening and found that the combined knockout of P2RY8 and GNAS can simultaneously achieve synergistic enhancement of “relieving migration inhibition” and “resisting functional inhibition, “providing direct evidence for multi-target gene editing strategies to improve CAR-T-cell infiltration into solid tumors” [130]. In terms of analytical techniques, intravital imaging provides a means to directly observe the dynamics of T-cell migration in vivo. Optical metabolic imaging utilizes the fluorescence and lifetime of nicotinamide adenine dinucleotide (phosphate) and flavin adenine dinucleotide coenzymes to achieve metabolic imaging at the single-cell level in living organisms [131]. Combining intravital multiphoton imaging with optical metabolic imaging enables simultaneous imaging of T-cell infiltration and metabolic trends. Label-free single-cell live imaging further reveals that T lymphocytes undergo rapid metabolic switching upon activation [132]. These technologies provide key tools for validating the effects of metabolic interventions on each trafficking stage.

Despite these encouraging advances, translating fundamental discoveries in metabolic reprogramming into clinical applications for regulating T-cell trafficking still faces multiple challenges. First, the broad and fundamental nature of metabolic pathways dictates that targeted metabolic interventions inevitably carry risks of off-target effects. Core metabolic pathways such as glycolysis, fatty acid oxidation, and the TCA cycle are ubiquitous across multiple cell types, and systemic intervention may affect the function of non-target cells. 2-DG interferes with T-cell function while inhibiting tumor metabolism, a contradiction repeatedly emphasized in multiple studies. Targeting glutamine metabolism similarly faces challenges arising from metabolic heterogeneity between tumor cells and immune cells. Second, there are significant differences in metabolic preferences between different T-cell subsets and even within the same subset in different tissue microenvironments, posing additional challenges for tissue-selective intervention. The finding that CB-839 has minimal effects on T-cell proliferation while significantly affecting tumor cell metabolism suggests that it is possible to achieve selective intervention by exploiting metabolic differences between tumor cells and T cells. However, the extent to which such selectivity can be generalized to other metabolic targets remains to be validated. Third, most direct evidence currently comes from in vitro experimental systems (e.g., three-dimensional collagen gels and transwell migration assays), with relatively limited validation in in vivo tumor and inflammation models. Although sirolimus-pretreated CAR T cells have shown enhanced intratumoral motility in xenograft models, translating these in vitro observed metabolic–migration coupling relationships into effective in vivo therapeutic strategies requires further study employing intravital imaging, competitive trafficking experiments, and residence kinetics analyses. Fourth, pharmacological parameters such as timing, dosage, and route of administration of metabolic interventions have not been systematically optimized. Although sirolimus can enhance the migratory capacity of CAR T cells, its inhibition of mTOR-dependent glycolysis also reduces the in vitro expansion rate of cells. How to achieve a balance between promoting migration and maintaining proliferation is a key issue that requires further exploration.

**Figure 6:**
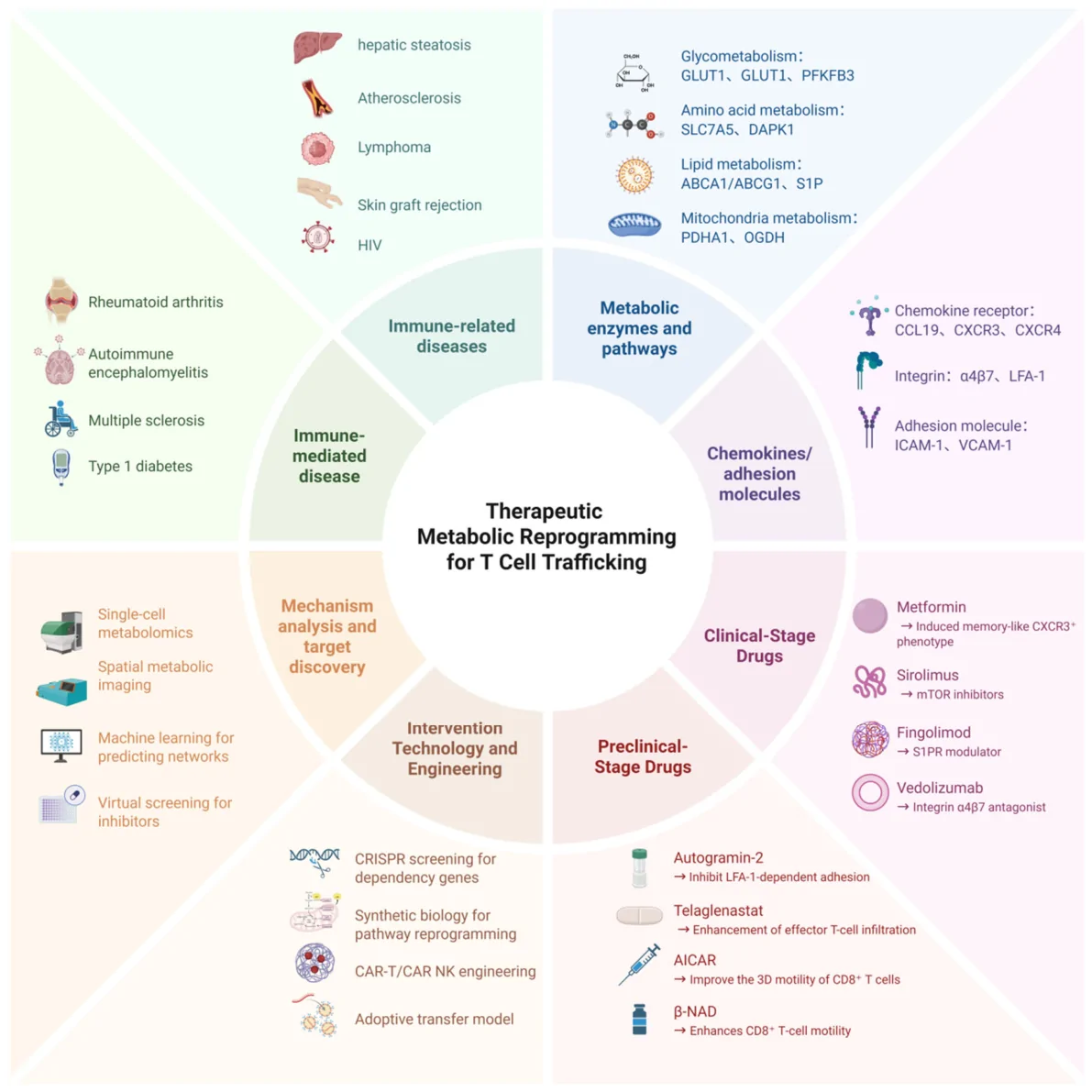
Therapeutic strategies targeting metabolic reprogramming to regulate T-cell trafficking.

In summary, the regulation of T-cell trafficking by metabolic reprogramming is highly unevenly distributed across the four metabolic types and four trafficking stages. Mitochondrial metabolism has the most robust evidence at the interstitial motility stage, while evidence at the rolling and firm adhesion stages is generally weak. At the translational level, integrin blockers and S1P receptor modulators that target trafficking receptors themselves have achieved significant clinical success, whereas strategies to regulate T-cell trafficking by directly targeting metabolic pathways are at a critical juncture of translation from preclinical to clinical settings. Drugs such as metformin, sirolimus, (2-DG), and CB-839 have shown potential in preclinical and early clinical studies to regulate T-cell migration, but face core obstacles including off-target effects, insufficient subset selectivity, and optimization of pharmacological parameters. Importantly, the clinical experience with CB-839/telaglenastat—particularly the negative phase II CANTATA trial—serves as a cautionary example that promising preclinical metabolic interventions may not translate into patient benefit without rigorous biomarker-driven trial design [126]. Emerging technologies such as CRISPR screening for metabolite-sensing receptors, gene editing to improve CAR-T-cell infiltration, and intravital metabolic imaging are providing new directions for breakthroughs in this field. The metabolic mechanisms by which non-antigen-specific T-cell subsets and costimulatory and coinhibitory receptors regulate trafficking, T-cell selectivity is optimized for metabolic inhibitors, and fundamental discoveries translate to clinical applications constitute the key directions for future research in this field. (as summarized in Figure 6).

This figure illustrates the therapeutic strategy of treating diverse diseases—including autoimmune disorders, cancer, metabolic diseases, and transplant rejection—by modulating metabolic reprogramming in T cells to control their trafficking to specific tissues. Core targets include key metabolic enzymes, homing-associated receptors/adhesion molecules, and signaling nodes. Emerging technologies such as single-cell metabolomics and Clustered Regularly Interspaced Short Palindromic Repeat (CRISPR) gene editing have advanced this field, as exemplified by preclinical and clinical agents like Fingolimod, Vedolizumab, and Metformin, highlighting the promise of this precision immunotherapy approach.

**Table 2:** Targeted drugs regulate metabolic reprogramming to influence T-cell trafficking.

Targeted Drugs	Target	Target Cell	Indication	Development Stage	Direct Measurement of Migration Outcome	Major Translational Limitations	References
Natalizumab	α4 integrin	Pathogenic effector T cells	Relapsing-remitting multiple sclerosis	Clinically approved	YES	Risk of progressive multifocal leukoencephalopathy accompanied by systemic immunosuppression	[133]
Vedolizumab	α4β7	T cells	Crohn’s disease; ulcerative colitis	Clinically approved	YES	May cause primary glomerulonephritis	[134,135]
Fingolimod	S1P1	T cells	Relapsing-remitting multiple sclerosis	Clinically approved	YES	Disrupts mitochondrial structure, induces thymocyte apoptosis, and leads to infection	[136,137]

**Table 3:** Metabolic inhibitors targeting metabolic reprogramming to regulate T-cell trafficking.

Metabolic Inhibitor	Metabolic Target	Pathway Downstream Trafficking Readout	Target Cell	Indication	Development Stage	Direct Measurement of Migration Outcome	Major Translational Limitations	References
2-Deoxyglucose (2-DG)	Glycolytic pathway	PSGL-1	TEMRA and EM CD8^+^ T cells	Solid tumors	Preclinical	YES	Severely impairs T-cell proliferation and activation	[63,66]
Metformin	AMPK pathway	VCAM-1; CXCR6	CD8^+^ T cells	Type 2 diabetes; tumors	Approved for original indication; preclinical for migration regulation	YES	Efficacy depends on tumor microenvironmental metabolism	[125]
CB-839	Glutaminase	mTOR signaling activity	Cancer cells, T cells	Advanced renal cell carcinoma (CANTATA); breast cancer and other solid tumors	Phase II randomized (CANTATA; *n* = 444) completed; no PFS benefitl	NO	Glutamine metabolic heterogeneity between tumor and immune cells	[126,127,138]
Sirolimus	Mitochondrial oxidative phosphorylation	CXCL12; mTORC1	T cells, CAR-T cells	Transplant rejection, autoimmune diseases; solid tumor CAR-T therapy	Clinically approved	YES	Suppresses mTOR-dependent glycolysis and reduces in vitro expansion rate of CD8^+^CAR-T cells	[128]

## Data Availability

No new data were created or analyzed in this study. Data sharing is not applicable to this article.
